# Efficient Semitransparent Organic Solar Modules with Exceptional Diurnal Stability Through Asymmetric Interaction Induced by Symmetric Molecular Structure

**DOI:** 10.1002/anie.202424287

**Published:** 2025-04-18

**Authors:** Sangjin Yang, Xuexiang Huang, Yongjoon Cho, Sungmo Koo, Yanni Ouyang, Zhe Sun, Seonghun Jeong, Thi Le Huyen Mai, Wonjun Kim, Lian Zhong, Shanshan Chen, Chunfeng Zhang, Hee‐Seung Lee, Seong‐Jun Yoon, Lie Chen, Changduk Yang

**Affiliations:** ^1^ School of Energy and Chemical Engineering Ulsan National Institute of Science and Technology (UNIST) 50 UNIST‐gil, Ulju‐gun Ulsan 44919 South Korea; ^2^ College of Chemistry/Institute of Polymers and Energy Chemistry (IPEC) Nanchang University Nanchang 330031 China; ^3^ Department of Chemistry KAIST 291 Daehak‐ro, Yuseong‐gu Daejeon 34141 South Korea; ^4^ National Laboratory of Solid State Microstructures and School of Physics Nanjing University Nanjing 210093 China; ^5^ Graduate School of Carbon Neutrality Ulsan National Institute of Science and Technology (UNIST) 50 UNIST‐gil, Ulju‐gun Ulsan 44919 South Korea; ^6^ School of Polymer Science and Engineering Chonnam National University 77 Yongbong‐ro, Buk‐gu Gwangju 61186 South Korea; ^7^ Center for Multiscale Chiral Architectures (CMCA) KAIST 291 Daehak‐ro, Yuseong‐gu Daejeon 34141 South Korea; ^8^ College of Intelligent Manufacturing and Materials Engineering Gannan University of Science and Technology 156 Kejia Avenue Ganzhou Jiangxi 341000 China; ^9^ Department of Chemistry and Materials Research Center Northwestern University 2145 Sheridan Road Evanston IL 60208 USA; ^10^ School of Energy & Power Engineering MOE Key Laboratory of Low‐Grade Energy Utilization Technologies and Systems CQU‐NUS Renewable Energy Materials & Devices Joint Laboratory Chongqing University Chongqing 400044 China

**Keywords:** Asymmetric molecular interaction, Dipole moments, Diurnal stability, Self‐assembly, Semitransparent organic solar cells

## Abstract

The symmetry‐breaking design strategy of nonfullerene acceptor can improve the performance of semitransparent organic solar cells (ST‐OSCs). However, no report exists on the “asymmetric molecular interaction” induced by symmetric molecular structure in nonfullerene acceptors. Herein, we showcase that 2D fluorophenyl outer groups in symmetric 4FY promote dipole‐driven self‐assembly through asymmetric molecular interactions, resulting in a tighter packed structure than Y6 with the same symmetric geometry. Such unique properties lead to high‐performance layer‐by‐layer OSCs, accompanied by simultaneously reduced energy and recombination losses and improved charge‐related characteristics. ST‐OSCs based on PCE10‐2F/4FY achieve notable power conversion efficiency (PCE) of 10.81%, average visible transmittance of 45.43%, and light utilization efficiency (LUE) of 4.91%. Moreover, exceptional diurnal cycling stability is observed in the ST‐OSCs based on PCE10‐2F/4FY with much prolonged *T*_80_ up to 134 h, which is about 17 times greater than the reference PCE10‐2F/Y6. Lastly, we fabricate highly efficient semitransparent organic solar modules based on PCE10‐2F/4FY (active area of 18 cm^2^), which shows PCE of 6.78% and the highest LUE of 3.10% to date for all‐narrow bandgap semitransparent organic solar modules. This work demonstrates that asymmetry‐driven molecular interactions can be leveraged to fabricate large‐area ST‐OSCs that are efficient and stable under realistic operating conditions.

## Graphical abstract

The symmetric molecular structure of 4FY has been shown to induce an “asymmetric molecular interaction”, which promotes a higher degree of molecular packing than in Y6. The asymmetry‐driven molecular interactions can be leveraged to fabricate large‐area semitransparent organic solar modules that are efficient and stable under realistic operating conditions.

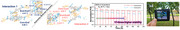

## Introduction

Semitransparent organic solar cells (ST‐OSCs) have been considered practical energy harvesting platforms for embedding into automobile‐, greenhouse‐, and building‐integrated photovoltaic devices.^[^ ^1^, ^2^, ^3^, ^4^, ^5^, ^6^, ^7^ ^]^ To realize its real‐world application, ST‐OSCs should meet multiple objectives in their high light utilization efficiency (LUE = power conversion efficiency (PCE) × average visible transmittance (AVT)), device stability, and large‐area processability.^[^ ^8^, ^9^, ^10^, ^11^, ^12^, ^13^ ^]^ Therefore, enormous research has sought to achieve such practical criteria through various approaches, including advances in material engineering, device architectures, and optical management in ST‐OSCs.^[^ ^14^, ^15^, ^16^, ^17^, ^18^, ^19^ ^]^

Meanwhile, the advent of near‐infrared (NIR)‐absorbing nonfullerene acceptor (NFA) Y6, which transmits the visible region (400−700 nm) and effectively leverages the (NIR) region (>700 nm), has marked a turning point in the advancement of ST‐OSCs by optimizing the trade‐off relationship between PCE and AVT.^[^ ^20^ ^]^ Especially, the unique intermolecular interaction of Y6 itself and the resultant optimized nanoscale morphology of active layers have not only dramatically upgraded the PCE of ST‐OSCs but also provided windows for improving device stability and large‐scale processability.^[^ ^21^, ^22^, ^23^, ^24^ ^]^ Therefore, various structural engineering strategies have emerged to modulate the intermolecular interaction of Y6‐based NFAs.^[^ ^25^, ^26^, ^27^, ^28^, ^29^, ^30^ ^]^

Among them, the “symmetry‐breaking” strategy in molecular structure has recently surfaced as an exceptionally effective approach to reinforce the intermolecular interaction of Y6‐based NFAs based on several bases: i) enhancing the molecular dipole moment,^[^ ^31^, ^32^, ^33^, ^34^, ^35^ ^]^ ii) generating far more diverse donor‒acceptor molecular interfacial structures,^[^ ^36^, ^37^ ^]^ and iii) reducing the charge‐carrier recombination.^[^ ^31^, ^38^, ^39^ ^]^ For instance, Luo et al. reported that side‐chain asymmetry in BTP‐PhC6‐C11 induced 3D charge transport networks with larger electronic couplings, leading to improved PCE of OSCs.^[^ ^35^ ^]^ Moreover, our group reported the NFA Y‐FIC‐γe, which possesses symmetry‐breaking end‐capping groups.^[^ ^32^ ^]^ Y‐FIC‐γe afforded tight molecular packing and optimized morphology because of its high dipole moment originated by molecular asymmetry, allowing the achievement of enhanced PCE, and thermal stability of OSCs simultaneously.^[^ ^32^ ^]^ However, the symmetry‐breaking molecular design strategy has unavoidable extended synthetic steps and more complicated purification processes vis‐à‐vis symmetric ones, which can be a bottleneck to mass production (Table S1). Consequently, developing NFAs, which have simultaneously the synthetic convenience of symmetric structures and the favorable intermolecular interactions of asymmetric structures, can provide a groundbreaking paradigm for efficient ST‐OSCs.

In this contribution, we confirm the first to reveal unique “asymmetric molecular interactions” of symmetric Y6‐based NFA 4FY through its single crystal and packing structure. In our previous report, 4FY with 2D fluorophenyl outer groups exhibited a high dipole moment and tighter‐range stacking structure than in Y6.^[^ ^40^ ^]^ In this study, we further revealed that the symmetric molecule 4FY has an asymmetric molecular conformation driven by two competitive intramolecular interactions (F⋯H and F⋯S interactions at the left‐side and right‐side 2D fluorophenyl outer groups), allowing tight molecular packing. Inspired by 4FY's exceptional molecular interaction features and our continued interest in high‐performance ST‐OSCs based on narrow‐bandgap (NBG) active layer materials, herein, we employed 4FY in all‐NBG active layer systems. Here, considering better thermally robust morphology with efficient vertical phase separation of the layer‐by‐layer (LBL) processing compared to BHJ one, we fabricated the LBL OSCs in this study. The distinctive packing of 4FY resulted in low recombination loss, low energy loss (*E*_loss_), and efficient charge separation and transport properties, with devices based on 4FY exhibiting excellent performance. An opaque OSC based on PCE10‐2F/4FY exhibited a PCE of up to 14.80%, and further, its ST‐OSC showcased a notable light utilization efficiency (LUE) of 4.91% along with a high PCE of 10.81% and AVT of 45.43%. More excitingly, this device also exhibited a *T*_80_ of 134 h, which is substantially longer than that of the reference PCE10‐2F/Y6 (*T*_80_ of 8 h) in the diurnal cycling stability test using the ISOS‐LC‐2I protocol. Lastly, we fabricated a highly efficient semitransparent organic solar module (ST‐OSM) based on PCE10‐2F/4FY (active area of 18 cm^2^), that showed a PCE of 6.78% and the highest LUE value of 3.10% to date for all‐NBG ST‐OSMs without complex optical engineering. Thus, the present study highlights that enhancing asymmetry molecular interactions for Y6‐based NFAs can ensure both improved photovoltaic performance and stability of large‐area ST‐OSCs.

## Results and Discussion

**Figure 1:**
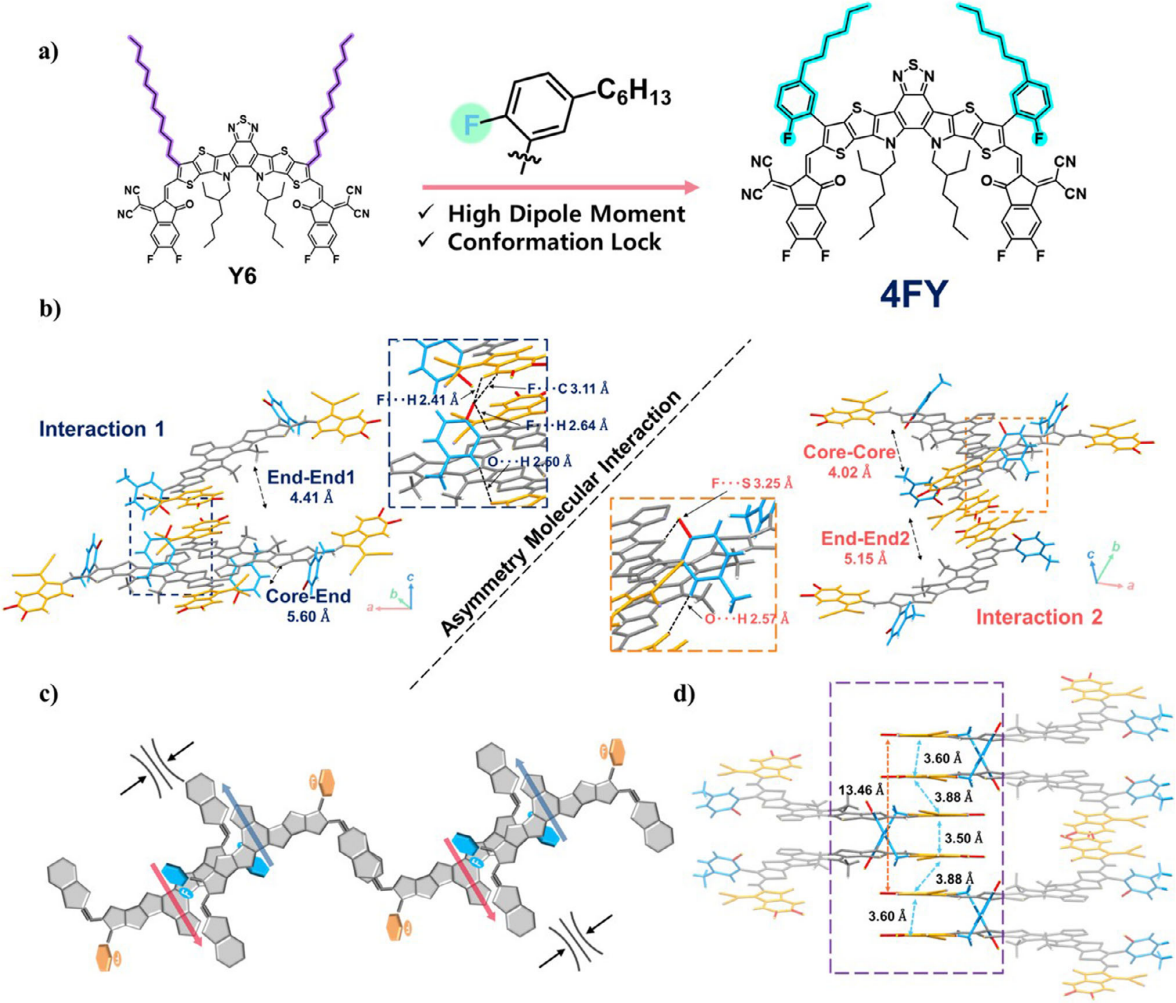
a) The chemical structures of 4FY and Y6, b) two types of crystal lattices with asymmetric interaction of 4FY, c) illustration of dipole‐driven self‐assembly structures of 4FY, and d) the distances of 4FY end groups packing in single crystallographic structures.

We conducted single‐crystal X‐ray diffraction (SCXRD) of 4FY to determine its single‐crystal and packing structures. Detailed single‐crystal data are provided in Table S2, and single‐crystal data for Y6 were extracted from previously reported data (Deposition Number 2006203).^[^ ^21^ ^]^ Deposition Number 2352496 (for 4FY) contains the supplementary crystallographic data for this paper. This data is provided free of charge by the joint Cambridge Crystallographic Data Centre and Fachinformationszentrum Karlsruhe Access Structures service. Figure 1a depicts the chemical structures of 4FY and Y6. As shown in Figure S1, with respect to the backbone core plane, the single crystal of 4FY exhibits a curved molecular geometry with large dihedral angles of the 2D fluorophenyl outer groups (53.7° for the left side and 59.4° for the right side) and small twist angles (0.7° for the left side and 1.7° for the right side) of end groups in comparison to Y6 with linear outer groups. These observations are correlated with the different intramolecular forces (e.g., F⋯H (distance of 2.64 Å) and F⋯S (distance of 3.25 Å) interactions) at the left‐side and right‐side 2D fluorophenyl outer groups, respectively.^[^ ^41^, ^42^, ^43^, ^44^ ^]^ Eventually, the different intramolecular interactions induced by fluorine atoms in 2D fluorophenyl outer groups resulted in different stretching conformation (diagonal/horizontal direction) of the 2D outer side chains, which means the asymmetric conformation in symmetric molecular structure. The crystal packing structure of 4FY exhibits a honeycomb shape and is similar to that of previously reported Y6‐based NFAs (Figure S2a), with lattice dimensions of 24.13 Å (corresponding to the stacking of the backbone cores), 57.03 Å (corresponding to the distance between the adjacent molecules), and 13.46 Å (corresponding to the stacking of end groups) along the *a*‐, *b*‐, and *c*‐axis directions, respectively. To be more specific, the 4FY consists of four stacking modes: two end−end types, one of which has a U shape with 4.41 Å (referred to as end−end1) and the other (referred to as end−end2) has an S shape with 5.15 Å, core−end type (5.60 Å), and core−core type (4.02 Å) (Figure S2b). Here, two types of crystal lattices are formed as combinations of two modes, specifically, end−end1/core−end and end−end2/core−core. The former (left side in Figure 1b) exhibits F⋯H (2.64 and 2.41 Å), F⋯C (3.11 Å), and O⋯H (2.50 Å) interactions, whereas the latter (right side in Figure 1b) shows the F⋯S (3.25 Å) and O⋯H (2.57 Å) bonding interactions. Consequently, such asymmetric molecular interactions in 4FY enable dipole‐driven self‐assembly structure and tighter‐range packing structure in the unit cells (3.60, 3.88, 3.50, 3.88, and 13.46 Å) compared to the previously well‐documented single crystal of Y6 (4.05, 4.30, 4.16, 4.30, and 20.08 Å) (Figures 1c,d, S2c). This could be the major factor explaining better PCEs of devices based on 4FY (*vide infra*).

Figure S3 shows the ultraviolet–visible (UV–vis) absorption spectra of PCE10‐2F, Y6, and 4FY. The PCE10‐2F structure is provided in Figure S3. The absorption spectra of dilute chloroform solutions of Y6 and 4FY are almost identical, with a maximum absorption centered at ∼730 nm. Upon transitioning from solution to thin films, the absorption spectrum of 4FY broadens, and the maximum absorption peak red‐shifts to 811 nm, which is ∼17 nm shorter in wavelength (optical bandgap of 1.38 eV) than that of Y6 (optical bandgap of 1.34 eV). This was ascribed to different solid‐phase aggregation caused by 2D fluorophenyl outer groups in 4FY. Both Y6 and 4FY films match well with the absorption‐complementary PCE10‐2F donor showing the maximum absorption at 705 nm and the optical band gap of 1.57 eV. A comparison of absorption strength reveals the higher absorption coefficient of the 4FY solution (117.6 M^−1^ cm^−1^) for the main peak compared to that of the Y6 solution (97.3 M^−1^ cm^−1^); at the same time, an opposite trend is observed for the corresponding films (Figure S3). These observations could be related to varied electrostatic interaction and molecular packing of 4FY induced by 2D fluorophenyl units. The energy‐level arrangement diagram determined via cyclic voltammetry shows that the highest occupied molecular orbital (HOMO) and lowest unoccupied molecular orbital (LUMO) energy levels in 4FY are up‐shifted by 50 meV compared to those in Y6 (Figure S5). Table S3 provides a detailed comparison of the photophysical properties of Y6 and 4FY. Density functional theory (DFT) calculations of Y6 and 4FY were performed using the B3LYP functional and 6–31G* basis set to confirm the observed HOMO and LUMO energy levels (Figures S6, S7).^[^ ^45^, ^46^, ^47^ ^]^ Additionally, the introduction of 2D fluorophenyl outer groups reinforces the dipole moment of 4FY and is thereby expected to promote dipole‐driven self‐assembly, increasing the degree of order at the donor−acceptor interface.

We used the LBL method (as described in the OSC fabrication part) to deposit PCE10‐2F/Y6 and PCE10‐2F/4FY films. The UV–vis absorption spectra of PCE10‐2F/Y6 and PCE10‐2F/4FY blend films (Figure S8) show that the blends exhibit wide spectral absorption but minimal overlap with the photopic response of the human eye (400−700 nm). Consequently, the blend films possess high transmittance, with AVT values over 58% (Table 1).

**Table 1:** Operating characteristics of opaque OSC devices under simulated AM 1.5 G, 100 mW cm^−2^ illumination.

Device	*V_OC_* (V)	*J_SC_* (mA cm^−2^)	*J_SC cal_* (mA/ cm^−2^)	FF (%)	PCE_max_ (%)	AVT of active layer (%)
PCE10‐2F/Y6	0.792	26.32	25.26	68.58	14.32	58.24
PCE10‐2F/4FY	0.817	25.20	24.06	72.06	14.80	58.36

**Figure 2:**
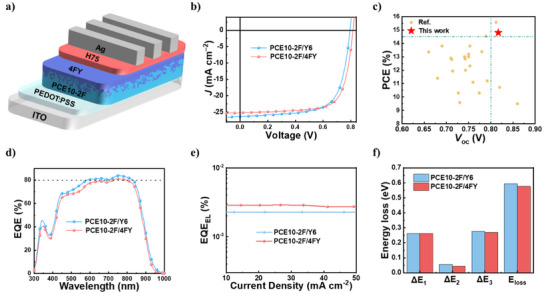
a) Schematic structure of opaque device, b) *J*‐*V* curves of the optimized opaque OSCs. c) PCE and VOC reported in the literatures (all‐NBG system). d) EQE curves of the optimized opaque OSCs. e) EQE_EL_, f) summary of energy loss diagrams for devices based on PCE10‐2F/Y6 and PCE10‐2F/4FY.

To evaluate the photovoltaic performances of PCE10‐2F/Y6 and PCE10‐2F/4FY systems, we fabricated opaque OSCs with the conventional structure (ITO/PEDOT:PSS/active layer/H75/Ag), as depicted in Figure 2a. The active layers were prepared using the LBL method based on the results of our previous work on PCE10‐2F.^[^ ^7^ ^]^ Each OSC was fully optimized by controlling parameters such as donor/acceptor (D/A) thickness, solvent, solvent additives, and thermal annealing. Detailed optimization conditions are provided in the Experimental Section and Supporting Information (Figure S9 and Table S4). The current density–voltage (*J*–*V*) characteristics of the OSCs with optimal D/A thickness (60/40 nm) under AM 1.5 G simulated solar illumination are shown in Figure 2b, and the relevant photovoltaic parameters are summarized in Table 1. The optimal reference device based on PCE10‐2F/Y6 exhibits a PCE of 14.32% with a *V*_OC_ of 0.793 V, a short circuit current density (*J*_SC_) of 26.32 mA cm^−2^, and a fill factor (FF) of 68.58%, values comparable with those in the previous report. At the same time, the device based on PCE10‐2F/4FY shows a higher PCE (14.80%) with significantly improved *V*_OC_ (0.817 V) and FF (72.06%) but slightly reduced *J*_SC_ (25.22 mA cm^−2^). Additionally, the *V*_OC_ of 0.817 V is approximately equal to those obtained for OSCs based on wide‐bandgap PM6, referred to as the standard of state‐of‐the‐art photovoltaic devices (Figure 2c and Table S5). The external quantum efficiency (EQE) spectra of the optimal OSCs are shown in Figure 2d. Owing to the strong near‐infrared absorption of the NBG donor and acceptor, all three devices exhibit ultrahigh EQEs (>80%) at 700–900 nm. The *J*_SC_ values obtained through EQE integration agree well with those measured from *J*–*V* curves within a mismatch of less than 5%, confirming the reliability of the experimental results. We quantitatively analyzed the *E*_loss_ of the optimal opaque devices (D/A = 60/40 nm) of PCE10‐2F/Y6 and PCE10‐2F/4FY, by measuring EQE and electroluminescence quantum efficiency (EQE_EL_; Figure 2e, Figure S10, and Table S6). The *E*_loss_ is the sum of Δ*E*_1_ (inevitable radiative energy loss above the bandgap (*E*_g_), Δ*E*_2_ (additional radiative energy loss below the *E*_g_), and Δ*E*_3_ (nonradiative energy loss); further details are provided in measurement in Supporting Information. The *E*_g_s values extracted from the respective EQE curves are 1.387 (PCE10‐2F/Y6) and 1.393 eV (PCE10‐2F/4FY), respectively (Figure S10). Although PCE10‐2F/Y6 and PCE10‐2F/4FY show identical Δ*E*_1_ (0.261 eV), their Δ*E*_2_ values are 0.056 and 0.046 eV, respectively. Their Δ*E*_3_ values, determined from the EQE_EL_ images (Figure 2e), decrease in the following order: PCE10‐2F/Y6 (0.277 eV) > PCE10‐2F/4FY (0.270 eV). Pertinent details are given in Figure 2f and Table S6. These results confirm the reduced radiative and nonradiative energy losses in the devices based on 4FY. As the energy losses are strongly related to charge transport and recombination behaviors, we measured space‐charge‐limited current hole and electron mobilities (*µ*_h_ and *µ*_e_) as well as the light intensity (*P*_light_) dependence of *J*_SC_ and *V*_OC_ of the three devices. The device based on PCE10‐2F/4FY exhibit better charge transport properties, with higher *µ*_h_ and *µ*_e_ and a more balanced *µ*_h_/ *µ*_e_ ratios (*µ*_h_ = 1.013 × 10^−3^ cm^2^ V^−1^s^−1^, *µ*_e_ = 9.721 × 10^−4^ cm^2^ V^−1^s^−1^, and *µ*_h_/ *µ*_e_ = 1.042 cm^2^ V^−1^s^−1^) compared with those of PCE10‐2F/Y6 (*µ*_h_ = 8.729 × 10^−4^ cm^2^ V^−1^s^−1^, *µ*_e_ = 8.211 × 10^−4^ cm^2^ V^−1^s^−1^, and *µ*_h_/ *µ*_e_ = 1.063 cm^2^ V^−1^s^−1^; Figure S11 and Table S7). As shown in Figure S12a, α and slope n(*kT*/*q*) can be quantified using *J*_SC_ ∝ *P*_light_^α^ equation in the log*J_sc_*–log*P*_light_ plot and *V*_OC_ ∝ n(*kT*/*q*)ln(*P*_light_) equation in the *V*_OC _− ln*P*_light_ plot, respectively, where *k*, *T*, and *q* are the Boltzmann constant, temperature (K), and charge, respectively. All the devices exhibit almost identical α values (∼0.99), but the devices based on PCE10‐2F/4FY show somewhat smaller n slope values (1.03 *kT*/*q*) than PCE10‐2F/Y6 (1.07 *kT*/*q*). These results imply that the trap‐assisted recombination loss that occurred in all the devices is slightly more suppressed in the devices based on PCE10‐2F/4FY. We also investigated the dependence of photocurrent density (*J*_ph_) on the effective voltage (*V*_eff_), transient photovoltage (TPV), and transient photocurrent (TPC) of the devices to elucidate charge‐carrier dynamics. The *J*_ph_−*V*_eff_ curves in Figure S12b indicate that the devices based on PCE10‐2F/4FY exhibit higher charge collection probabilities (*P*_coll_) than PCE10‐2F/Y6, even despite their lower exciton dissociation (*P*_diss_) probabilities. TPV and TPC curves suggest longer decay lifetimes of the devices based on PCE10‐2F/4FY compared to that of PCE10‐2F/Y6, and similar extraction times (of ∼0.3 *µs* (Figure S12c, S12d). Collectively, these findings could be attributed to the tight‐range packing structure centered on the end group. They help explain the improved *V*_OC_ and FF in the devices based on PCE10‐2F/4FY, where highly efficient charge collection with less recombination losses occurs, even though the driving force for exciton dissociation is smaller.^[^ ^48^, ^49^ ^]^

Since there is a compromise between light transmittance and PCE, a prerequisite for achieving efficient ST‐OSCs, as our goal in this study is optimizing the active‐layer thickness. Thereby, we attempted to adjust the active layer thickness of the PCE10‐2F/4FY system to find a good point of balance between PCE and AVT (Figures S9, S13). A decrease in the thickness of the D‐layer with a higher visible absorption would increase the AVT while resulting in a certain reduction in PCE, a sharp decline in FF, a slight decrease in *J*_SC_, and almost no changes in *V*_OC_. At the same time, any change in the thickness of the optimal 40 nm A‐layer negatively affects all the photovoltaic parameters (Table S4). Consequently, the optimal trade‐off between PCE and AVT for all devices is the D/A thickness of 45/40 nm, with the corresponding *J*–*V* and EQE curves of the devices presented in Figure S13 and Table S8. At the D/A thickness of 45/40 nm, the device based on PCE10‐2F/4FY exhibits higher PCE and AVT than the reference device based on PCE10‐2F/Y6.

**Figure 3:**
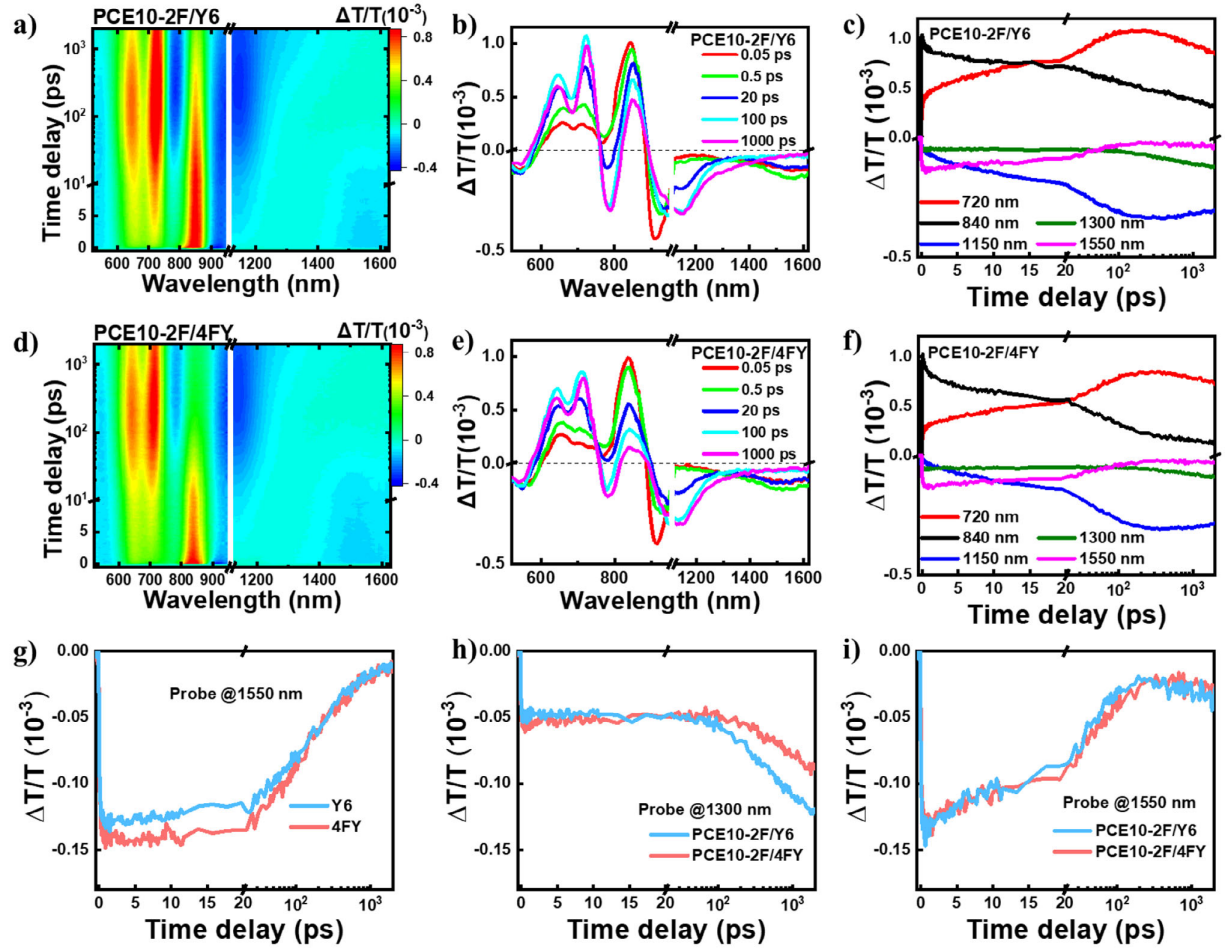
a) and d) TA data recorded from PCE10‐2F/Y6 and PCE10‐2F/4FY LBL film of 850 nm excitation, respectively. b) and e) TA spectra of the PCE10‐2F/Y6 and PCE10‐2F/4FY LBL film at different delay times, respectively. c) and f) TA dynamics at different wavelengths in the PCE10‐2F/Y6 and PCE10‐2F/4FY LBL film, respectively. g) TA dynamics of i‐EX states at 1550 nm in the neat Y6 and 4FY films and h) the PCE10‐2F/Y6 and PCE10‐2F/4FY LBL films. i) TA dynamics of the formation of low‐lying triplet excited states observed at 1300 nm in the PCE10‐2F/Y6 and PCE10‐2F/4FY LBL films.

We performed transient absorption (TA) spectroscopy to gain deeper insight into the charge generation and recombination processes in the LBL active layer films (Figure 3a,d). By selectively exciting the acceptor layer (Y6 and/or 4FY), we track the pure hole transfer process. Upon excitation, the ground bleaching (GSB) signal of the acceptor layer around 840 nm emerges in accordance with the absorption spectra (Figure 3b,e). The formation of intramoiety excited (i‐EX) states occurs due to intermolecular interactions within the acceptor layer, resulting in the presence of a well‐defined absorption band around 1550 nm in both the LBL films and neat acceptor films. As depicted in Figure 3g, the stronger formation of i‐EX states in the neat 4FY film indicates a more densely packed structure compared to the neat Y6 film. In the LBL films, the i‐EX states undergo further hole transfer to facilitate the charge generation process, leading to a significantly shortened lifetime of 34.5 ps for PCE10‐2F/Y6 and 38.3 ps for PCE10‐2F/4FY (∼183.6 ps for both neat acceptor films) as shown in Figure 3h. With the ongoing hole transfer, the GSB signal of the donor layer around 720 nm and an excited‐state absorption (ESA) band around 1150 nm rise, denoting the formation of charge separation states (Figure 3c,f). Afterward, the signal of charge separation states begins to decay at hundreds of picoseconds, accompanied by the emergence of a broad ESA band centered at 1300 nm. This observation implies the onset of charge recombination and the formation of low‐lying triplet excited states. The less formation of triplet excited states in PCE10‐2F/4FY (Figure 3i) may be attributed to the compact packing of 4FY and a decreased mixing at the D‐A interface.^[^ ^50^, ^51^ ^]^

The thin‐film morphology of the LBL blend active layers was investigated using atomic force microscopy (AFM) and transmission electron microscopy (TEM). Compared to PCE10‐2F/Y6 (root mean square (RMS) roughness of 2.28 nm), films based on PCE10‐2F/4FY exhibit relatively smooth top surfaces with lower RMS roughness values (1.43 nm), as shown in AFM images (Figure S14). Additionally, slightly larger aggregates are observed in the TEM images of PCE10‐2F/Y6 (Figure S14). We also characterized molecular ordering/packing characteristics of individual D‐ and A‐layers and LBL films using grazing incidence wide‐angle X‐ray scattering (Figures S15, S16); detailed crystallographic parameters are listed in Table S9.

As previously reported, the PCE10‐2F‐only and Y6‐only films exhibit a face‐on preferential orientation, along with strong out‐of‐plane π–π stacking peaks, *q*_z_ = 1.597 Å^−1^ (*d*‐spacing = 3.936 Å) and *q*_z_ = 1.703 Å^−1^ (*d*‐spacing = 3.689 Å) for PCE10‐2F and Y6, respectively, and in‐plane peaks, *q*_xy_ = 0.282 Å^−1^ (*d*‐spacing = 22.313 Å) and *q*_xy_ = 0.288 Å^−1^ (*d*‐spacing = 21.797 Å) for PCE10‐2F and Y6, respectively. At the same time, the 4FY‐only film shows slightly shorter *d*‐spacings (3.599 and 21.18 Å) even despite its similar face‐on dominant orientation, suggesting a higher packing density, which agrees well with the SCXRD data presented above. LBL processing further reduced the *d*‐spacings, with the reduction for LBL films based on PCE10‐2F/4FY being higher than that for PCE10‐2F/Y6 (Figure S16), indicating the more compact and ordered molecular packing structures of LBL films based on 4FY, explaining the efficient charge‐related properties, as discussed above.

**Figure 4:**
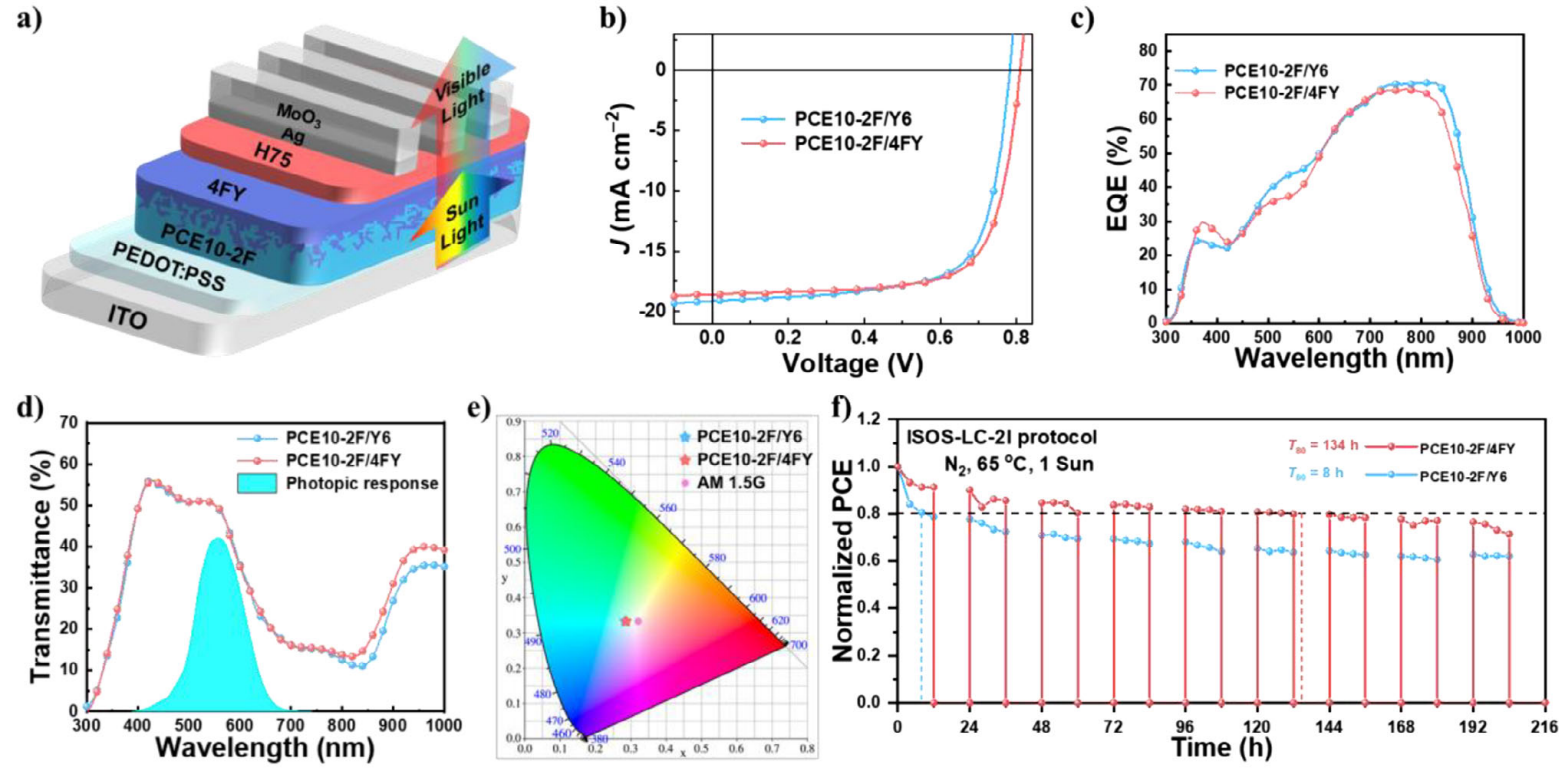
a) Schematic structure of semitransparent devices, b) *J*–*V* curves, c) EQE, and d) transmittance curves of ST‐OSCs. e) Color coordinates of ST‐OSC devices on a CIE 1931xy chromaticity diagram. f) Diurnal stability test (ISOS‐LC‐2I protocol) of ST‐OSC devices.

Considering the well‐balanced PCE and AVT at the D/A thickness of 45/40 nm, we fabricated full ST‐OSC devices with the optimized D/A thickness and a transparent Ag/MoO3 cathode (thickness of 15/35 nm), as shown in Figure 4a. The *J*–*V* curves, EQE spectra, and transmittance spectra of the optimal ST‐OSCs are exhibited in Figure 4b–d. The transmission spectra of the electrodes are shown in Figures S17a,b, 4d present the reflection and transmission spectra of the full ST‐OSCs, respectively, indicating their good transmittance in the visible region.

The ST‐OSC based on PCE10‐2F/Y6 shows a PCE of 10.56% with a *J*_SC_ of 19.14 mA cm^−2^, a *V*_OC_ of 0.783 V, an FF of 70.33%, and an AVT of 45.46%, yielding an LUE of 4.80%. Benefiting from the significant increases in *V*_OC_ and FF, the ST‐OSC device based on PCE10‐2F/4FY exhibits a PCE of 10.81% with an AVT of 45.43%, yielding a promising LUE of 4.91%. Detailed device parameters are listed in Table 2. As shown in Figure 4c, the EQE spectra of the ST‐OSCs are more concentrated in the NIR region, and the responses in the visible light region are very weak. Additionally, quantum utilization efficiency (QUE) (QUE = EQE + T, where T is transmittance) was used to investigate the light energy utilization of the ST‐OSC devices. As shown in Figure S17c, the QUE values of all the ST‐OSCs are below 90% in the entire spectral region (300–1000 nm). These observations further confirm the high optical transmission of our ST‐OSCs.

**Table 2:** Operating characteristics of semitransparent devices under simulated AM 1.5 G, 100 mW cm^−2^ illumination.

Device		*V*_OC_ (V)	*J*_SC_ (mA cm^−2^)	FF (%)	PCE^a)^ (%)	AVT^a)^ (%)	LUE^a)^ (%)
Small area	PCE10‐2F/Y6	0.783	19.14	70.33	10.56(10.33)	45.46(43.80)	4.80(4.52)
	PCE10‐2F/4FY	0.808	18.58	72.02	10.81(10.52)	45.43(44.04)	4.91(4.63)
Module	PCE10‐2F/4FY	3.92	2.48	69.69	6.78	45.43	3.10

^a)^ The values obtained from five independent devices.

Commission Internationale de l'Eclairage (CIE) 1931 color space, color rendering index, and correlated color temperature are also important parameters for evaluating the overall characteristics of ST‐OSC devices. These parameters accurately describe the color perception and fidelity of ST‐OSCs under an AM 1.5 G lighting source and can be calculated from the transmission spectra of ST‐OSCs. The color coordinates for PCE10‐2F/Y6 and PCE10‐2F/4FY are (0.288,0.335) and (0.287,0.335), respectively. Their associated color rendering indices are 78.3 and 78.9, and correlated color temperatures are 7883 and 7945 K, respectively. The corresponding CIE coordinates are plotted in Figure 4e. The CIE coordinates of the ST‐OSCs are close to the AM 1.5 G solar spectrum point (0.332, 0.343) and the white point (0.333, 0.333), indicating neutral color characteristics.

Furthermore, we conducted diurnal cycling stability tests of the unencapsulated ST‐OSCs under practical operating conditions. In these tests, the devices were subjected to the diurnal cycle for 24 h (1 d) according to the ISOS‐LC‐2I protocol (1 sun at 65 °C for 12 h (day) and darkness at room temperature for 12 h (night); Figure 4f).^[^ ^52^ ^]^ After only 1 day, a rapid drop in PCEs is observed for PCE10‐2F/Y6, accompanied by a dramatic burn‐in loss with *T*_80_ = 8 h, where *T*_80_ is the time at which the PCE of the device decreases to 80% of its initial value. In contrast, the ST‐OSCs based on PCE10‐2F/4FY exhibit a small PCE decay with significantly weaker burn‐in (about only 10% after 1 d), resulting in substantially longer *T*_80_ lifetimes of 134 h for PCE10‐2F/4FY. Theoretically, device degradation, including burn‐in, occurs because of morphological changes in the structure of the active layers. Thus, we further conducted AFM and TEM analyses to investigate the morphology of the active layer in both the systems before and after the diurnal cycling stability tests (Figure S18). The RMS values of both PCE10‐2F/4FY and PCE10‐2F/Y6 LBL semitransparent films increase to some extent after 134 h, as seen from their height images. Scrutiny of AFM phase and TEM images reveals that after 134 h, there is minimal detectable change in the PCE10‐2F/4FY semitransparent LBL film, whereas the growth of larger and coarser phase domains is visualized, suggesting the superior morphological stability of PCE10‐2F/Y6 semitransparent LBL film. In addition to the tighter‐range molecular packing, the enhanced multisecondary bonding interactions, including π−π stacking and van der Waals interactions, effectively inhibit molecular movements under external stress.^[^ ^53^, ^54^, ^55^, ^56^ ^]^ Therefore, the A‐layer in 4FY possesses superior morphological stability, and its interdiffusion into the D‐layer induces a more robust pseudo‐D/A‐layer configuration.

**Figure 5:**
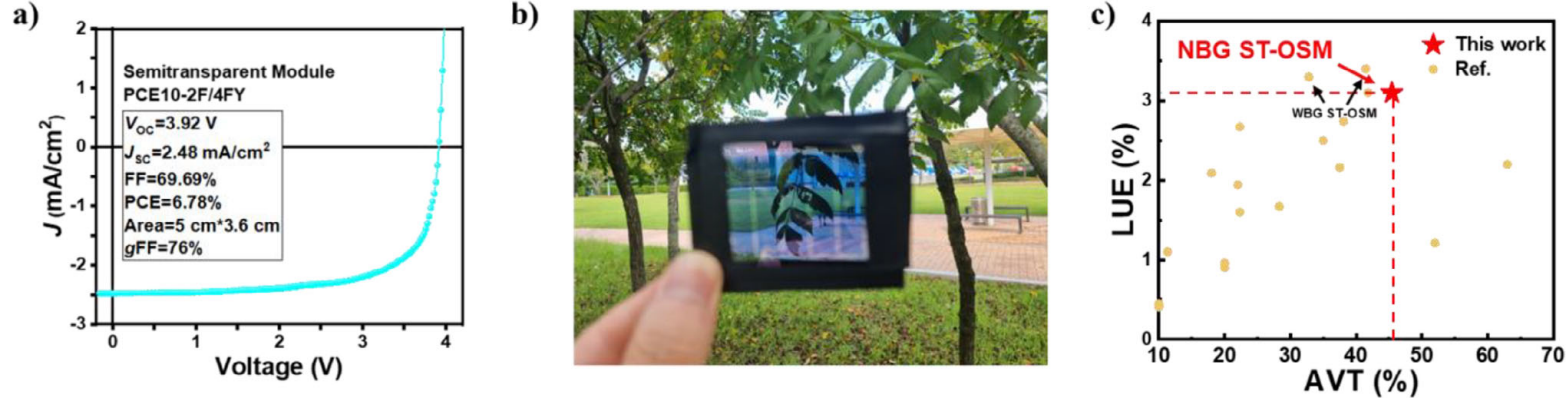
a) *J*–*V* curves of ST‐OSM based on PCE10‐2F/4FY. b) A campus photo with ST‐OSM. c) AVT and LUE values reported in the literature for the ST‐OSMs.

As photovoltaic modules represent an essential test bed for real‐world applications, we finally evaluated PCE10‐2F/4FY in large‐area modules. The 5‐series connected ST‐OSM based on PCE10‐2F/4FY (active area of 18 cm^2^) is constructed with the identical structure of ST‐OSCs as discussed above (Figure S19), and its *J*–*V* curve of the optimal ST‐OSM is provided in Figure 5a. Figure 5b shows a photo of better‐performing ST‐OSM based on PCE10‐2F/4FY placed in front of our campus landscape, confirming excellent aesthetics and transparency. This module showcased a PCE of 6.78% with a *J*_SC_ of 2.48 mA cm^−2^, a *V*_OC_ of 3.92 V, an FF of 69.69%, and a geometrical FF of 76%, yielding the highest LUE value of 3.10% reported to date for all‐NBG ST‐OSMs without complex optical control (Figure 5c, Table 2, and S10). Thus, the utilization of 4FY is a promising strategy for fabricating ST‐OSMs that are efficient and stable under practical operating conditions.

## Conclusion

In summary, through an in‐depth investigation into the molecular structure of 4FY, we discovered that 2D fluorophenyl outer groups within it regulate the asymmetric molecular interactions and promote dipole‐driven self‐assembly, resulting in its higher degree of molecular packing than in Y6. This simultaneous decrease in *E*_loss_ and recombination loss led to efficient charge separation and transport, enabling the high performance of LBL OSCs based on 4FY. In particular, the OSC based on PCE10‐2F/4FY afforded a PCE of 14.80%, and the full ST‐OSC based on PCE10‐2F/4FY showed an LUE of 4.91% accompanied by a PCE of 10.81% and an AVT of 45.43%. More importantly, the stability tests of the ST‐OSCs under diurnal cycling according to the ISOS‐LC‐2I protocol demonstrated excellent outdoor stability of the ST‐OSCs based on 4FY. Particularly, PCE10‐2F/4FY exhibited a *T*_80_ of 134 h, whereas reference PCE10‐2F/Y6 showed a *T*_80_ of 8 h. This increased stability was attributed to the superior morphological robustness induced by the unique crystal packing structure of 4FY. Finally, we fabricated a 5‐series connected ST‐OSM based on 4FY (active area of 18 cm^2^). The ST‐OSM based on PCE10‐2F/4FY exhibited the highest LUE value of 3.10% reported to date for all‐NBG ST‐OSMs accompanied by a PCE of 6.78% with a *J*_SC_ of 2.48 mA cm^−2^, a *V*_OC_ of 3.92 V, an FF of 69.69%, and geometrical FF of 76%. The knowledge gained from this study can directly contribute to the commercial scalability of large‐area ST‐OSCs, potentially revolutionizing the solar power industry.

## Conflict of Interests

The authors declare no conflict of interest.

## Supporting information



Supporting Information

Supporting Information

## Data Availability

The data that support the findings of this study are available from the corresponding author upon reasonable request.
