# Supplementary material for: Efficient Semitransparent Organic Solar Modules with Exceptional Diurnal Stability Through Asymmetric Interaction Induced by Symmetric Molecular Structure
Source: Angew Chem Int Ed Engl. 2025 Apr 18;64(24):e202424287. doi: 10.1002/anie.202424287 (PMC12144883; doi:10.1002/anie.202424287)
Supplement: Supplementary file 1 — Supporting Information [file ANIE-64-e202424287-s001.docx]

Efficient Semitransparent Organic Solar Modules with Exceptional Diurnal Stability through Asymmetric Interaction Induced by Symmetric Molecular Structure

*Sangjin Yang^a,§^, Xuexiang Huang^a,b,h,§^, Yongjoon Cho^a,i §^, Sungmo Koo^c,g^, Yanni Ouyang^d^, Zhe Sun^a^, Seonghun Jeong^a^, Thi Le Huyen Mai^a^, Wonjun Kim^a^, Lian Zhong^a^, Shanshan Chen^j^, Chunfeng Zhang^*d^, Hee-Seung Lee^*c,g^, Seong-Jun Yoon^*a,f^, Lie Chen^*b^, and Changduk Yang^*a,e^*

^a^School of Energy and Chemical Engineering, Perovtronics Research Center, Low Dimensional Carbon Materials Center, Ulsan National Institute of Science and Technology (UNIST), 50 UNIST-gil, Ulju-gun, Ulsan 44919, South Korea.

^b^College of Chemistry/Institute of Polymers and Energy Chemistry (IPEC), Nanchang University, Nanchang 330031, China.

^c^Department of Chemistry, KAIST, 291 Daehak-ro, Daejeon, 34141, South Korea.

^d^National Laboratory of Solid State Microstructures and School of Physics, Nanjing University, Nanjing 210093, China.

^e^Graduate School of Carbon Neutrality, Ulsan National Institute of Science and Technology (UNIST), 50 UNIST-gil, Ulju-gun, Ulsan 44919, South Korea.

^f^School of Polymer Science and Engineering, Chonnam National University, 77 Yongbong-ro, Buk-gu, Gwangju 61186, South Korea.

^g^Center for Multiscale Chiral Architectures (CMCA), KAIST, 291 Daehak-ro, Daejeon, 34141, South Korea.

^h^College of Intelligent Manufacturing and Materials Engineering, Gannan University of Science and Technology, 156 Kejia Avenue, Ganzhou 341000, Jiangxi, China.

^i^Department of Chemistry and Materials Research Center, Northwestern University, 2145 Sheridan Road, Evanston, IL 60208, USA.

^j^School of Energy & Power Engineering, MOE Key Laboratory of Low-Grade Energy Utilization Technologies and Systems, CQU-NUS Renewable Energy Materials & Devices Joint Laboratory, Chongqing University, Chongqing 400044, China.

**1.** **Materials and Synthesis**

PCE10-2F, Y6, 4FY, and H75 were synthesized according to previously reported methods. BDT-2F and F-TT were purchased from Derthon Optoelectronic Materials Science Technology Co LTD (Shenzhen, China) and 2-(5,6-difluoro-3-oxo-2,3-dihydro-1*H*-inden-1-ylidene)malononitrile was purchased from SunaTech Inc, and the other chemicals and reagents were purchased from Sigma-Aldrich, Tokyo Chemical Industry Co., Ltd, and Alfa Aesar Chemical Company and used without further purification.

2. Experimental Section

UV-Vis spectroscopy

Optical absorption spectra of the polymers were measured on a PerkinElmer model Lambda 900 UV-vis/near-IR spectrophotometer. For UV-Vis-NIR spectra of the 4FY, their solutions (4.00 mg mL^-1^ in CF) were used, and the films were prepared by deposition from the 4.00 mg mL^-1^ concentrated CF solution via the spin-casting method at 700 rpm. The optical bandgaps were estimated from the absorption onset of the as-cast thin films.

Cyclic voltammetry

CV measurements were performed on an Iviumstat.h with a three-electrode cell system in a nitrogen-bubbled 0.1 M tetra-*n*-butylammonium hexafluorophosphate (*n*-Bu_4_NPF_6_) solution in acetonitrile at a scan rate of 100 mV^−1^ s^−1^ at room temperature. An Ag/Ag^+^ electrode, platinum wire, and material-coated glassy carbon electrode were used as the reference electrode, counter electrode, and working electrode, respectively. The Ag/Ag^+^ reference electrode was calibrated using an Fc/Fc^+^ redox couple as an external standard, whose oxidation potential was set at −4.8 eV with respect to the zero-vacuum level. HOMO and LUMO energy levels of Y6 and 4FY were obtained from the equation HOMO (eV) = − (E_ox_^onset^ – E_Fc_^onset^ + 4.8) and LUMO (eV) = − (E_red_^onset^ − E_Fc_^onset^ + 4.8).

Energy loss details

The total energy loss (E_loss_) in organic solar cells (OSCs) can be roughly calculated using E_loss_ = Δ E_1_ + Δ E_2_ + Δ E_3_, which is divided into three parts: (1) Δ E_1_ = E_g_ − *V_OC_*_, SQ_, where E_g_ denotes the bandgap, is the maximum *V_OC_* under the Shockley–Queisser limit; Δ E_1_ is an unavoidable part, which is the energy loss due to absorption above the band gap; (2) Δ E_2_ = *V_OC_*_, SQ_ − *V_OC_*_, Rad_ is the energy loss caused by radiative recombination under the bandgap, which is the part affected by the reorganization energy and energy disorder degree of the blends; (3) Δ E_3_ is the voltage loss caused by non-radiative recombination, and it has a quantitative relationship with the luminescence efficiency of photovoltaic materials, which can be expressed as follows: Δ E_3_ = qΔ*V_OC_*_, non-rad_ = − kT ln(EQE_EL_) (k is Boltzmann’s constant, and T is room temperature)

Fabrication of Solar Cells

The opaque device was fabricated with ITO/PEDOT:PSS/active layer/H75/Ag traditional structure. The ITO-coated glass substrates were cleaned by ultrasound for 15 minutes in sequence in water/detergent, water, acetone, and isopropanol, and then treated in ultraviolet-ozone for 1200 seconds. The PEDOT:PSS solution was spin-coated on top of the cleaned ITO-coated glass substrate, and the PEDOT:PSS film thickness was approximately 25 nm (4000 rpm). After annealing at 150 °C for 20 min, then the substrates were transferred into a glove box. PCE10-2F (concentration of 8 mg mL^−1^ in CF) was spun onto the PEDOT: PSS layer at 2500 rpm (60 nm), 5500 rpm (45 nm) for 40 s. 1,8-diiodooctane (DIO) and 1-chloronaphthalene (CN) were added (volume ratio 0.25% and 0.25%, respectively) to CF solution (concentration of 10 mg mL^−1^) of Y6 and 4FY. Then, acceptors were spun onto the PCE10-2F layers at 2300 rpm (40 nm) for 40 s. After annealing at 100 °C for 10 min, the H75 was dissolved in methanol at 2 mg mL^−1^ and spin-coated on the active layer at 3000 rpm for 30 s. Finally, 100 nm thick Ag layers were deposited on the active layer under a high vacuum of ~3x10^−4^ Pa.

The semitransparent device was fabricated with ITO/PEDOT:PSS/active layer/H75/Ag/MoO_3_ structure. Before the cathode deposition process, the same procedures above were followed, with the excepting spin casting speed of the active layer (5500 rpm for PCE10-2F (45 nm) and 2300 rpm for acceptor (40 nm)). Next, 15nm thickness Ag and layers were deposited on the active layer under a high vacuum of ~3x10^−4^ Pa. Then, MoO_3_ (35 nm) was evaporated onto the surface of Ag. The overlapping area of the cathode and anode was 4 square millimeters. *J*–*V* curves of PCE10-2F/Y6 and PCE10-2F/4FY devices were measured under the standard AM 1.5G spectrum of 100 MW cm^−2^.

Fabrication of Solar Module

The structure of the module is identical to that of the small-area device described in the article. First, a PEDOT:PSS hole transport layer was applied onto the patterned ITO glass substrate (P1 channel) using the doctor blade coating method at a speed of 10 mm/s, followed by thermal annealing at 150°C for 10 minutes. Next, PCE10-2F and 4FY were successively deposited onto the GLASS/ITO/PEDOT:PSS substrate via doctor blade coating at a speed of 20 mm/s and a substrate temperature of 40°C, with undergoing thermal annealing at 100°C for 10 minutes. Finally, after the H75 electron transport layer was deposited by doctor blade coating at 5 mm/s, femtosecond laser patterning was carried out on the substrate (P2 channel). The prepared substrate was then transferred to a thermal vacuum evaporation system. First, a 100nm thick silver electrode was deposited using mask plate No. 1 (cover the P2 channel), followed by the deposition of a 15nm thin silver electrode using mask plate No. 2 (P3 channel) (Figure S19).

TA spectroscopy measurement

The picosecond-resolved TA measurements with pump wavelength at 850 nm were conducted using a Yb:KGW laser (Pharos, Light Conversion). The pump pulses were emitted from a home-built noncollinear optical parametric amplifier. The probe beam was supercontinuum by focusing a small fraction of the fundamental 1030 beam to a 5 mm sapphire plate for visible detection or a 5 mm YAG plate for infrared detection. A short pass filter (10SWF-1000-B, Newport) or a long pass filter (FELH1100, Thorlabs) was employed in the supercontinuum to exclude the fundamental beam for visible detection (520–950 nm) or infrared detection (1100–1620 nm), respectively. The supercontinuum light was split into two beams for balanced detection. The probe and reference beams were then routed to either a double-line Si camera (S14417, Hamamatsu) for visible detection or a double-line InGaAs camera (G11608, Hamamatsu) for infrared detection. The cameras were mounted on a monochrometer (Acton 2358, Princeton Instrument). Pulse-to-pulse spectral analysis was conducted at 50 kHz for visible detection and 16 kHz for infrared detection using a homemade field-programmable gate array (FPGA) control board. The signal-to-noise ratio (ΔT/T) was better than 10-5 after averaging 25k pump-on and pump-off shots for each data point.

AFM and TEM measurements

AFM and TEM were measured via a Hitachi AFM5100N in the tapping mode and a JEOL USA JEM-2100F (Cs corrector) transmission electron microscope equipped with an energy-dispersive X-ray analysis at an acceleration voltage of 200 kV, respectively.

GIWAXS measurement

GIWAXS measurement was conducted at the PLS-II 9A beamline of the Pohang Accelerator Laboratory (PAL) in Korea. The X-rays coming from the in-vacuum undulator were monochromated (λ = 1.10994 Å) using a double crystal monochromator and focused both horizontally and vertically (450 (H) × 60 (V) *µ*m^2^ in FWHM (full width at half maximum) @ the sample position) using K–B type mirrors. The GIWAXS sample stage was equipped with a 7-axis motorized stage for the fine alignment of the sample, and the incidence angle of the X-ray beam was set to be 0.12° for the neat and blend films. The GIWAXS patterns were recorded with a 2D CCD detector (Rayonix SX165), and the X-ray irradiation time was 5–30 s, dependent on the saturation level of the detector. Diffraction angles were calibrated using a sucrose standard (monoclinic, P21, a = 10.8631 Å, b = 8.7044 Å, c = 7.7624 Å, and β = 102.938°) and the sample-to-detector distance was ≈231 mm. CCL was calculated according to the following Scherrer equation:

CCL = 2πK/Δq (Equation 1)

In this equation, CCL is the crystal coherence length, K is a shape factor (0.9), and Δq is the FWHM of a diffraction peak.

SCXRD measurement

A rhombohedral black crystal of 4FY was obtained by the slow solvent diffusion method using dibromomethane and methanol. X-ray diffraction data for the crystal were collected under a stream of N_2_ (g) at 100 K using monochromatic synchrotron radiation (λ = 0.7 Å) and Dectris Pilatus 6 M detector at the 11C beamline of the PAL. The data were indexed, integrated, and scaled using the HKL3000, and the hkl file was generated through XPREP. The structure was solved by direct methods with SHELXT, and refinement was performed using SHELXL embedded in Olex2. The disorder model was used to refine the alkyl chains from the fluorophenyl group but failed due to its ambiguous Fourier difference map. Therefore, those alkyl chains were treated by SQUEEZE embedded in Olex2 (solvent mask).

Diurnal operational stability measurement

Diurnal cycling stability tests of the unencapsulated ST-OSCs under ISOS-LC-2I protocol were conducted. ST-OSCs were subjected to 1 sun (LSH-7320 ABA LED Solar Simulator, ORIEL) at 65°C for 12 h (day) and darkness at room temperature for 12 h (night). The *J*–*V* characteristics of ST-OSCs under diurnal stability tests were measured at 4-hour intervals in the daytime (on a computer-controlled Keithley 2400 Source-Measure Unit.)

Calculation of AVT

The AVT is calculated using

$VLT=\frac{\int T(\lambda)P(\lambda)S(\lambda)d(\lambda)}{\int P(\lambda)S(\lambda)d(\lambda)}$ $VLT=\frac{\int T(\lambda)P(\lambda)S(\lambda)d(\lambda)}{\int P(\lambda)S(\lambda)d(\lambda)}$ $VLT=\frac{\int T(\lambda)P(\lambda)S(\lambda)d(\lambda)}{\int P(\lambda)S(\lambda)d(\lambda)}$ $VLT=\frac{\int T(\lambda)P(\lambda)S(\lambda)d(\lambda)}{\int P(\lambda)S(\lambda)d(\lambda)}$ $AVT=\frac{\int T(\lambda)V(\lambda)S(\lambda)d(\lambda)}{\int P(\lambda)S(\lambda)d(\lambda)}$ (Equation 2)

where λ is the wavelength, T is the transmission, V is the normalized photopic spectral response of the eye, and S is the solar photon ﬂux (AM1.5G). It is estimated by taking the average of the transparency of the devices in the visible region (380–740 nm) based on the photonic response of the human eye.


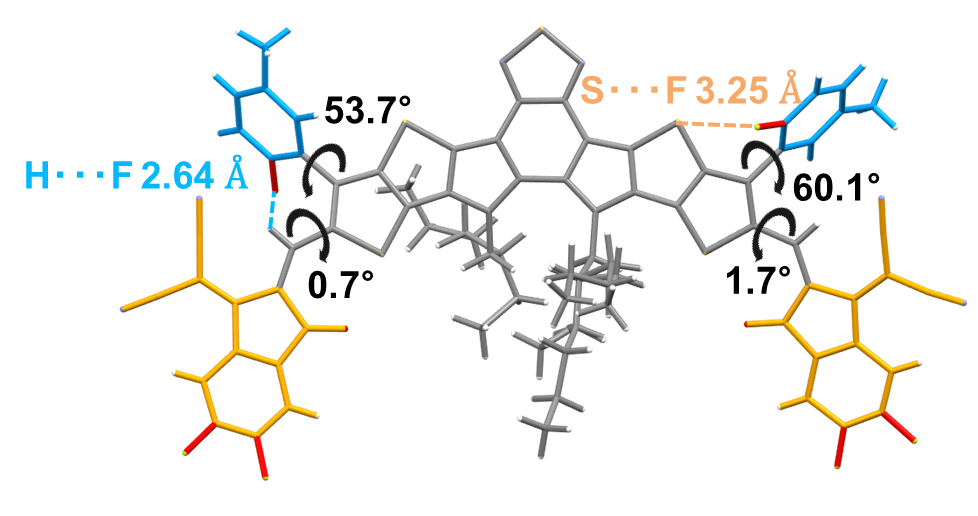


**Figure S1.** Molecular backbone structure of 4FY (dashed sky blue and light orange arrows represent the F···H and F···S, respectively) obtained by single crystal X-ray diffraction.

**
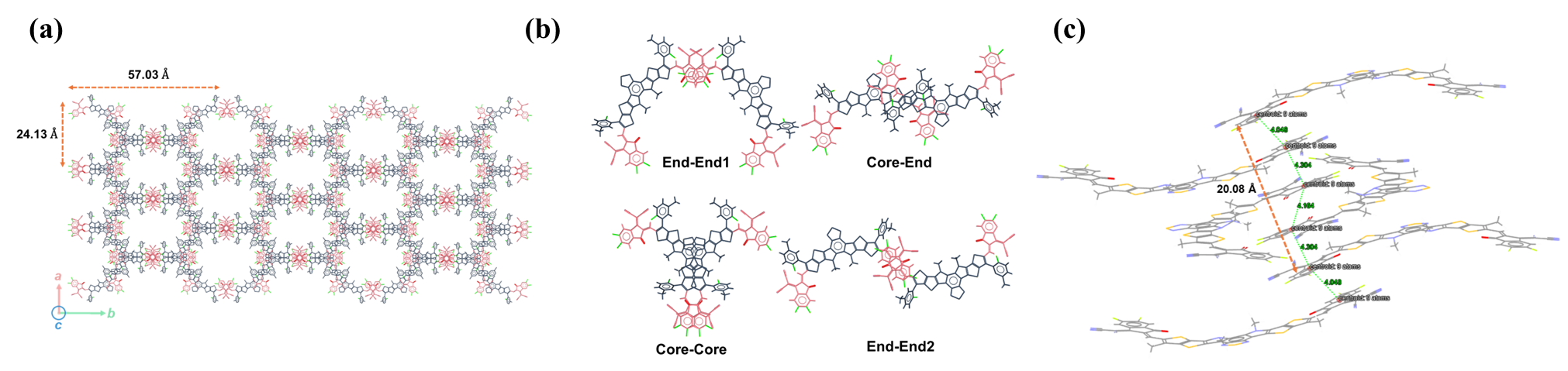
** **Figure S2.** (a) The 3D network packing of 4FY along *c*-axis and (b) the molecular stacking modes of dimer in single crystal network of 4FY. (c) The repeating distances of end packing in single crystal of Y6.


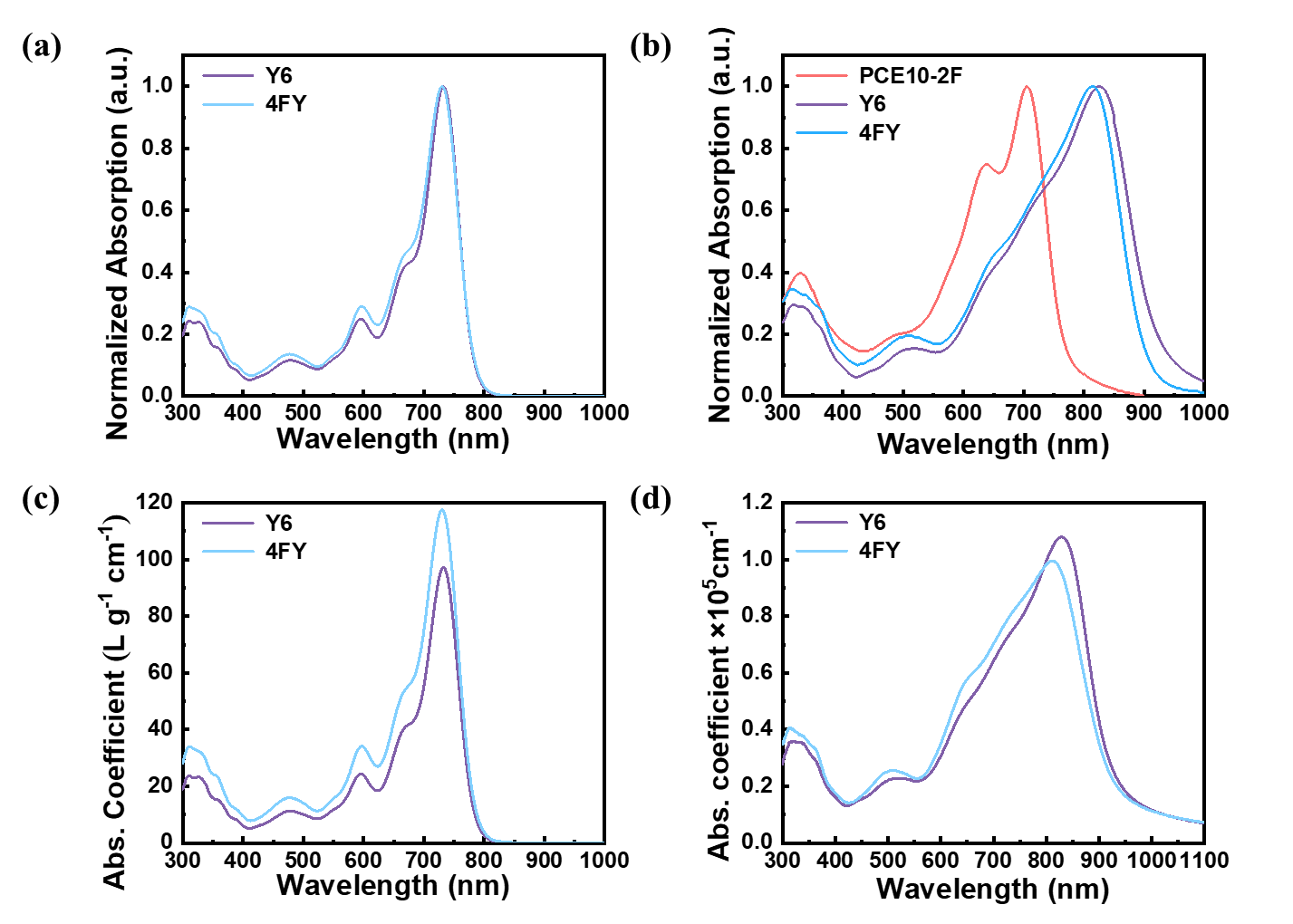


**Figure S3.** Normalized UV-vis absorption spectra of (a) Y6 and 4FY in CF solutions and (b) PCE10-2F. Y6, and 4FY in films. Absorption coefficients of Y6 and 4FY in (c) CF solutions and (d) films.

**Figure S4**. The chemical structure of PCE10-2F donor polymer.


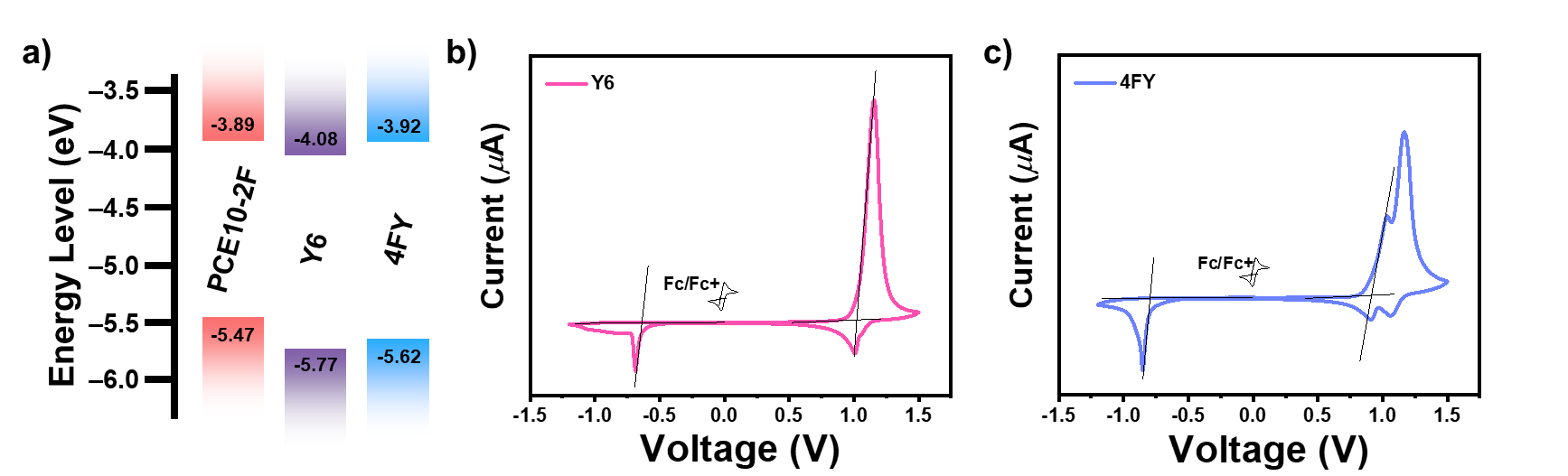


**Figure S5.** (a) Energy diagram of active materials and cyclic voltammetry curves of b) Y6 and c) 4FY.
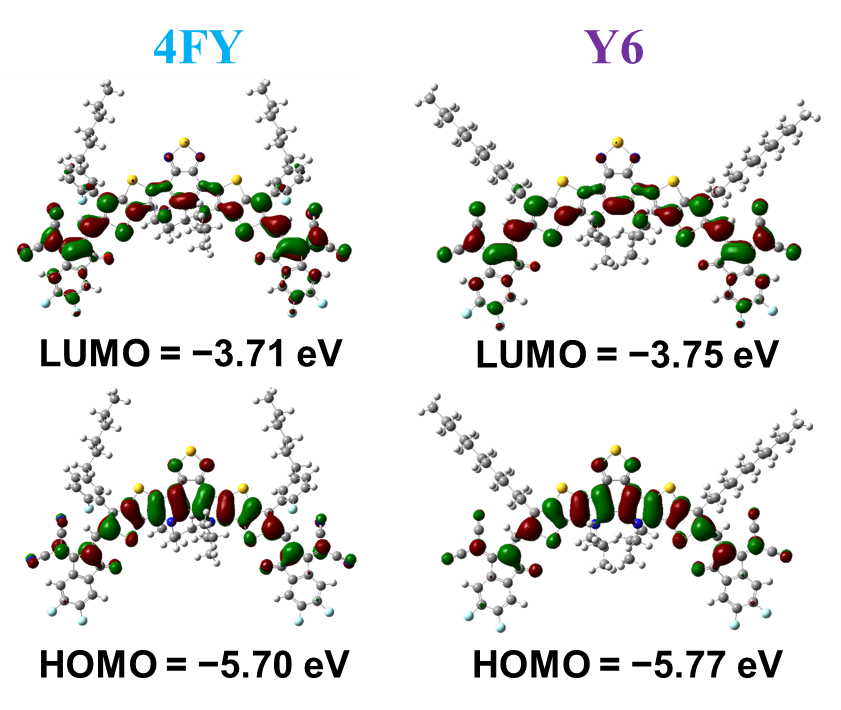


**Figure S6.** Distribution of HOMO and LUMO wave functions and calculated energy levels of 4FY and Y6.


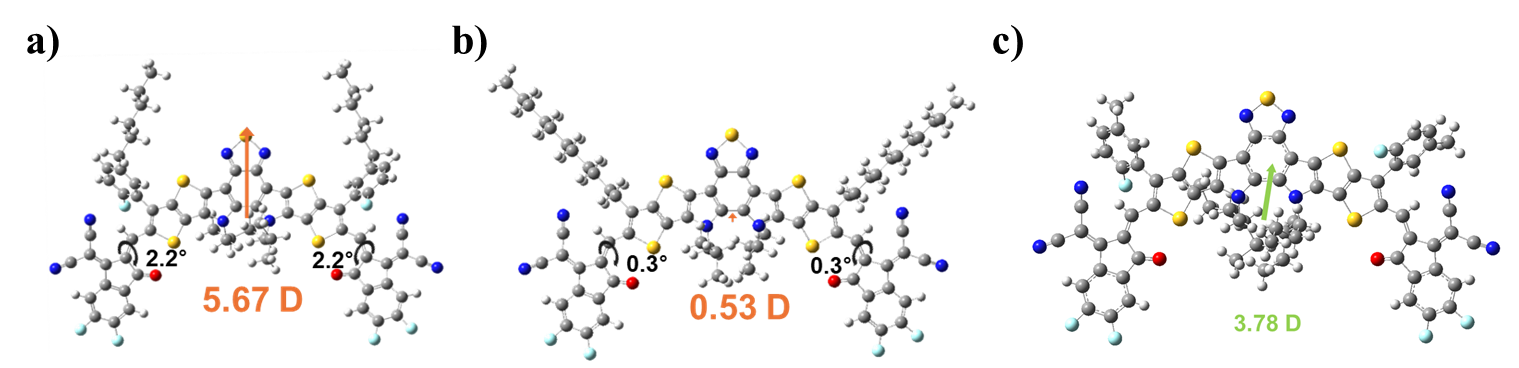


**Figure S7.** Optimized molecular geometries of a) 4FY and b) Y6 (orange solid arrows represent the total dipole moment of the whole molecule) and c) density functional theory calculation obtained by single crystal X-ray diffraction. (solid green arrow represent direction of dipole moment.


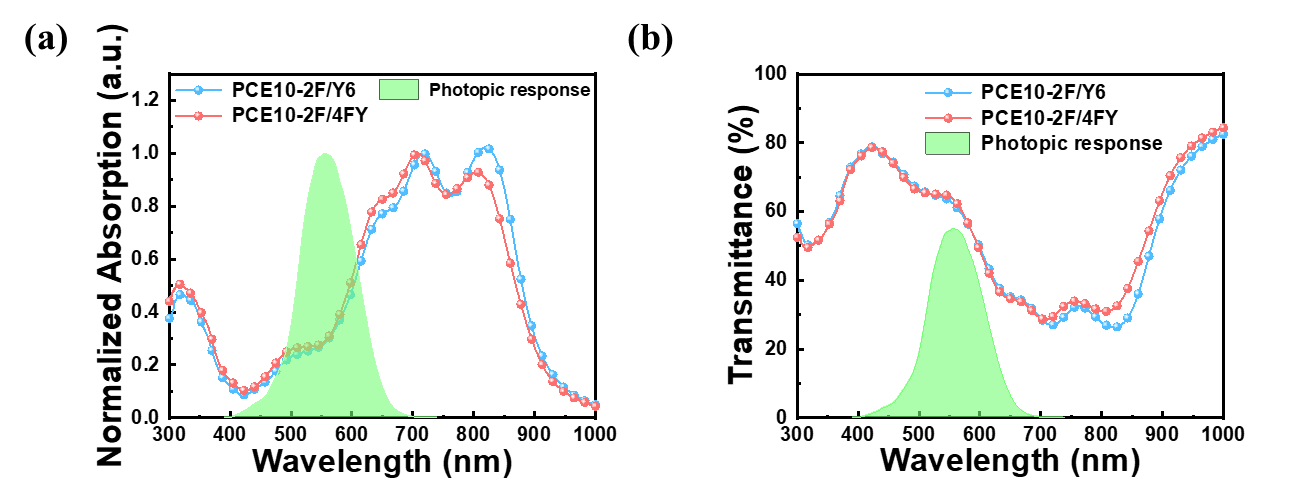


**Figure S8.** (a) Normalized UV-vis absorption spectra and (b) transmittance spectra of blend films.

**Figure S9.** *J*-*V* curves of PCE10-2F/4FY optimized opaque OSCs.


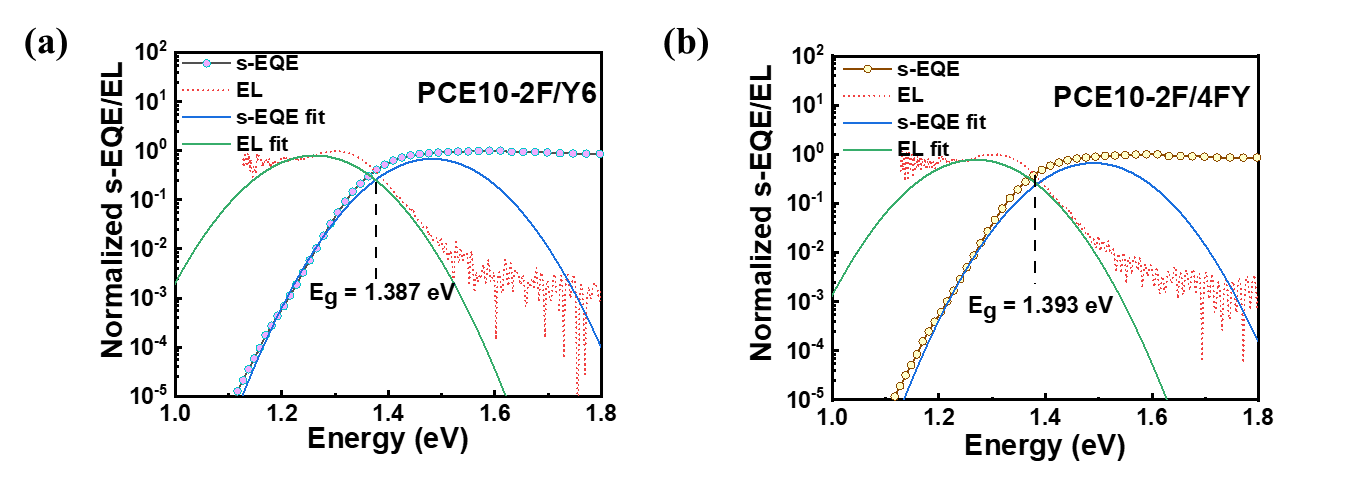


**Figure S10.** Normalized s-EQE and electroluminescence spectra of (a) PCE10-2F/Y6 and (b) PCE10-2F/4FY.


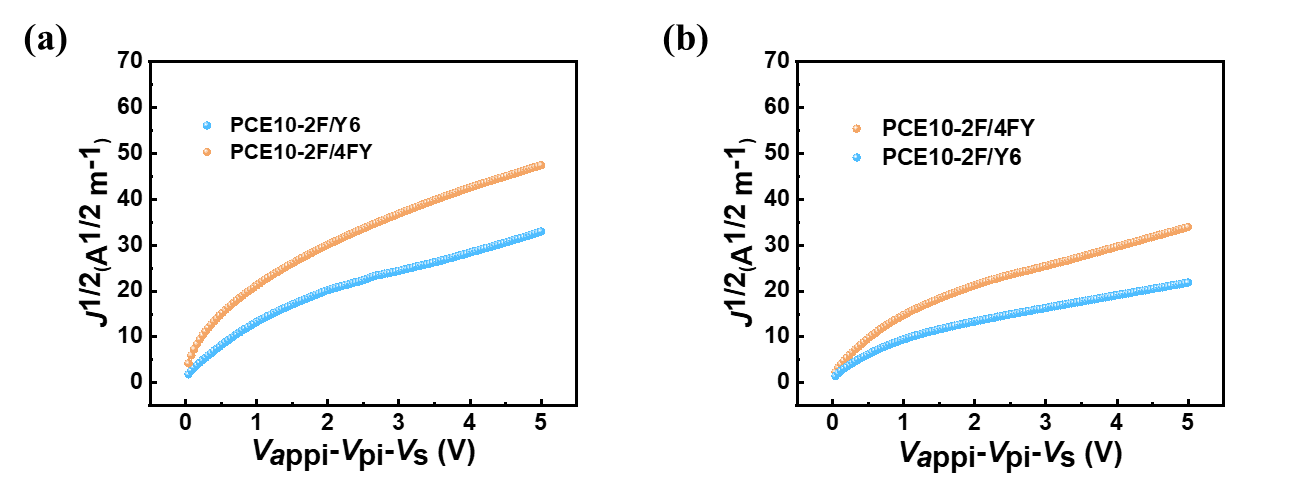


**Figure S11.** (a) *J*^1/2^−*V* plots of hole-only devices and (b) *J*^1/2^−*V* plots of electron-only devices (in dark).


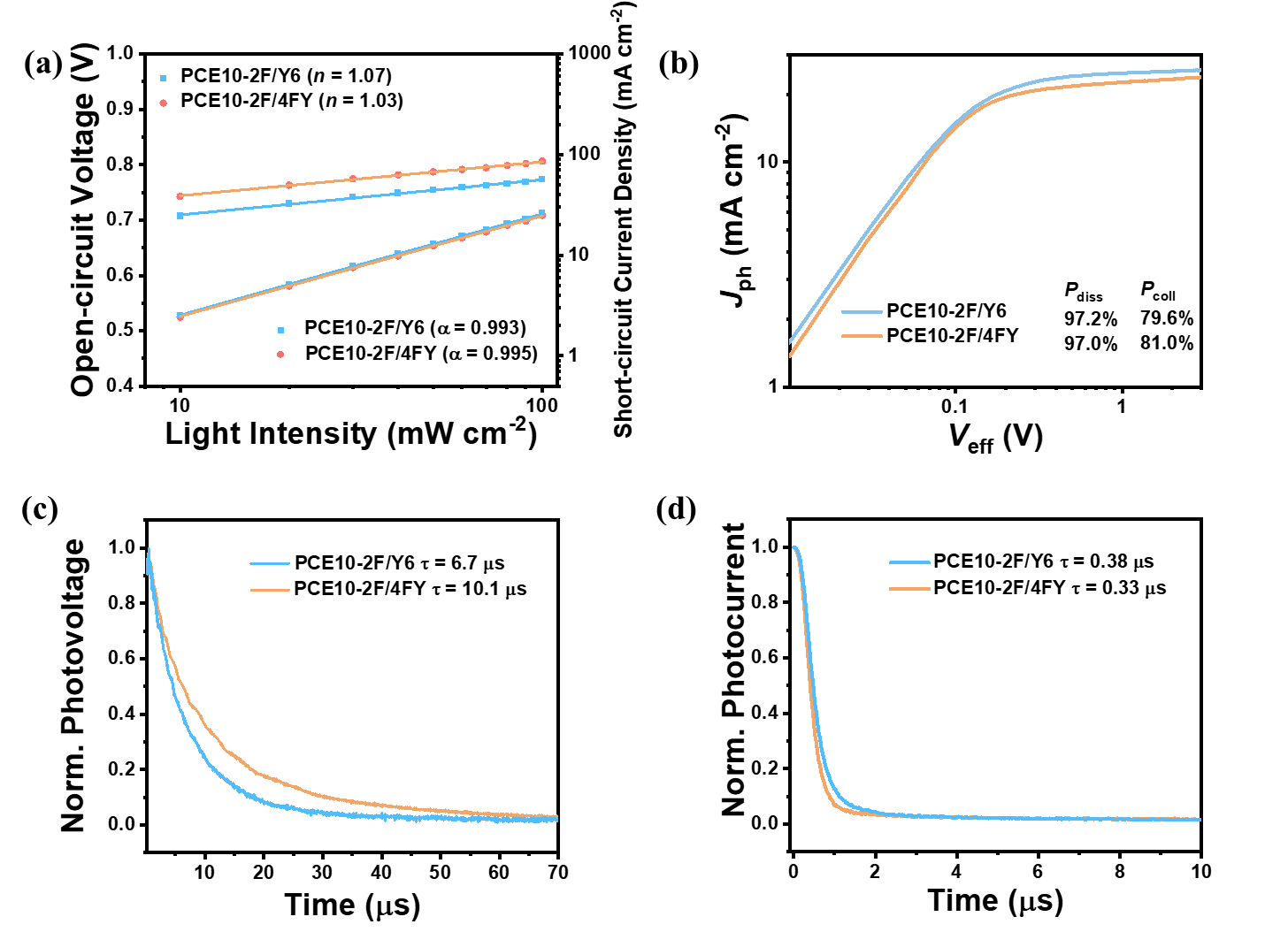


**Figure S12** (a) Dependencies of the open-circuit voltage/short-circuit current density on light intensity, (b) *J*_ph_−*V*_eff_ curves, (c) TPV, and (d) TPC curve of devices based on PCE10-2F/Y6 and PCE10-2F/4FY.


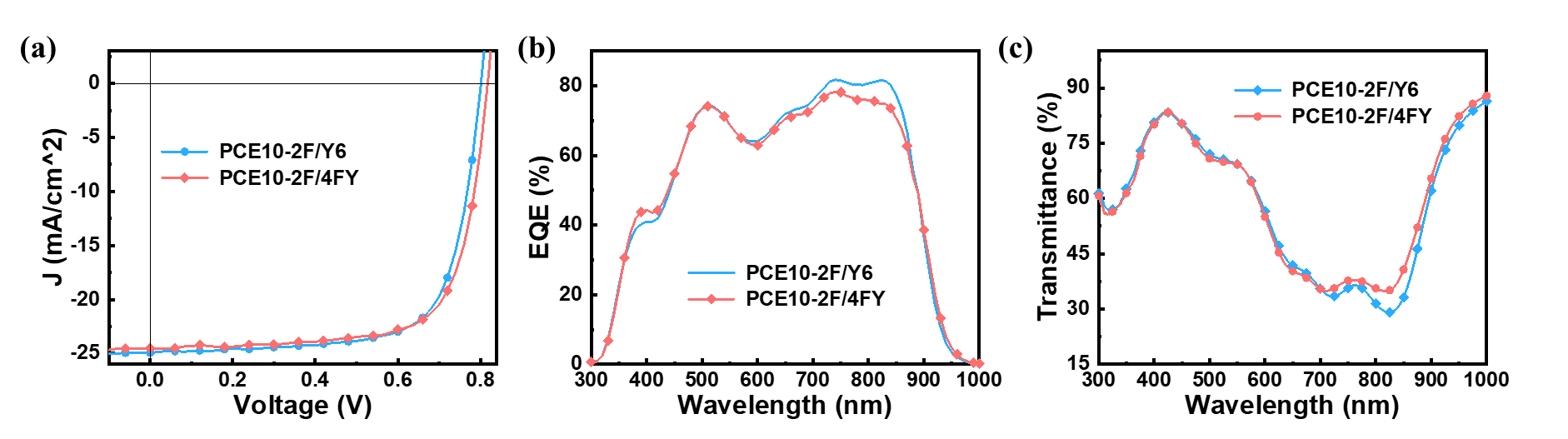


**Figure S13.** (a) *J*-*V*, (b) EQE, (c) transmittance curves of PCE10-2F/Y6 and PCE10-2F/4FY optimized opaque OSCs (D/A=45 nm/40 nm).


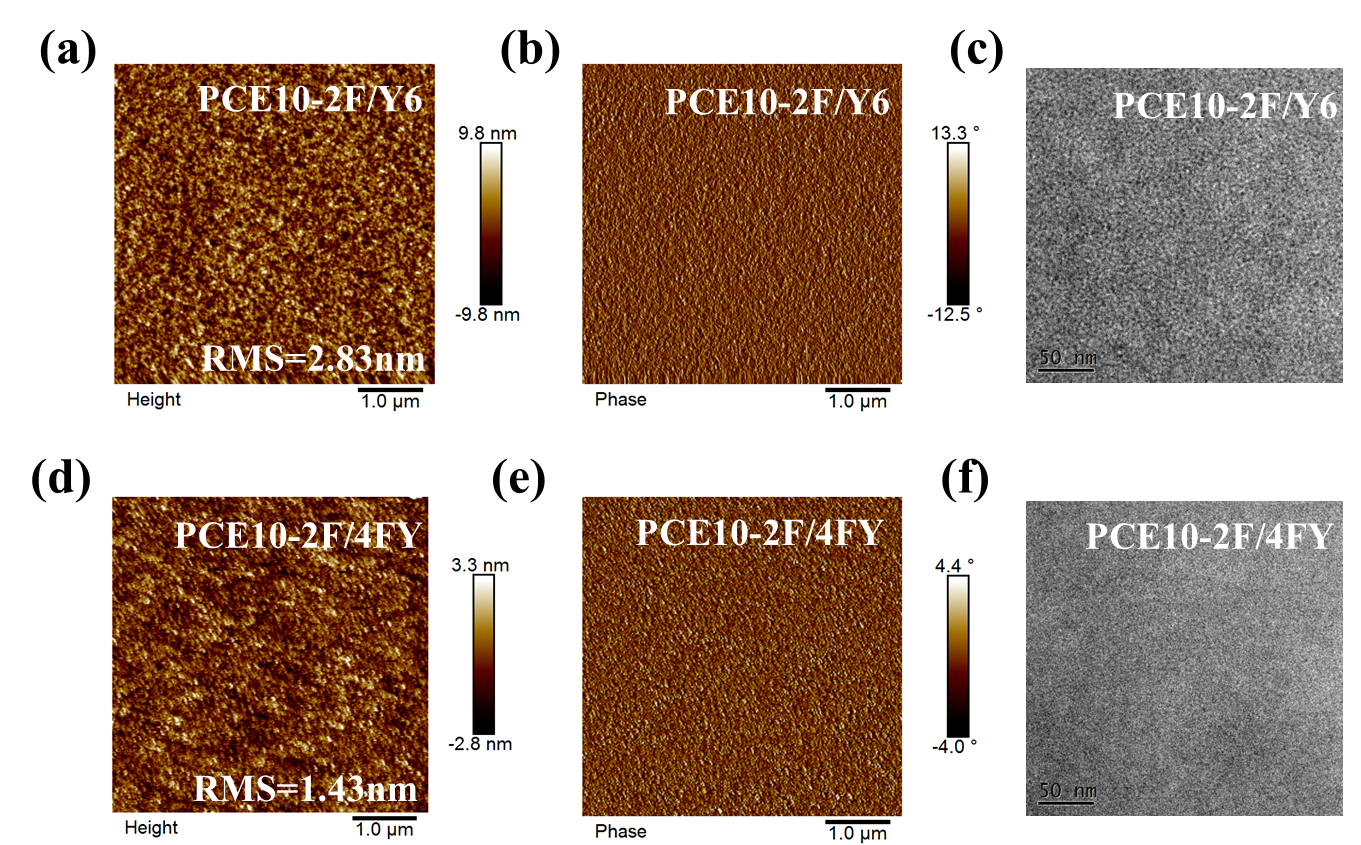


**Figure S14.** AFM surface height (a and d), phase (b and e), and TEM images (c and f) of optimized PCE10-2F/Y6 and PCE10-2F/4FY blend films, respectively.

**
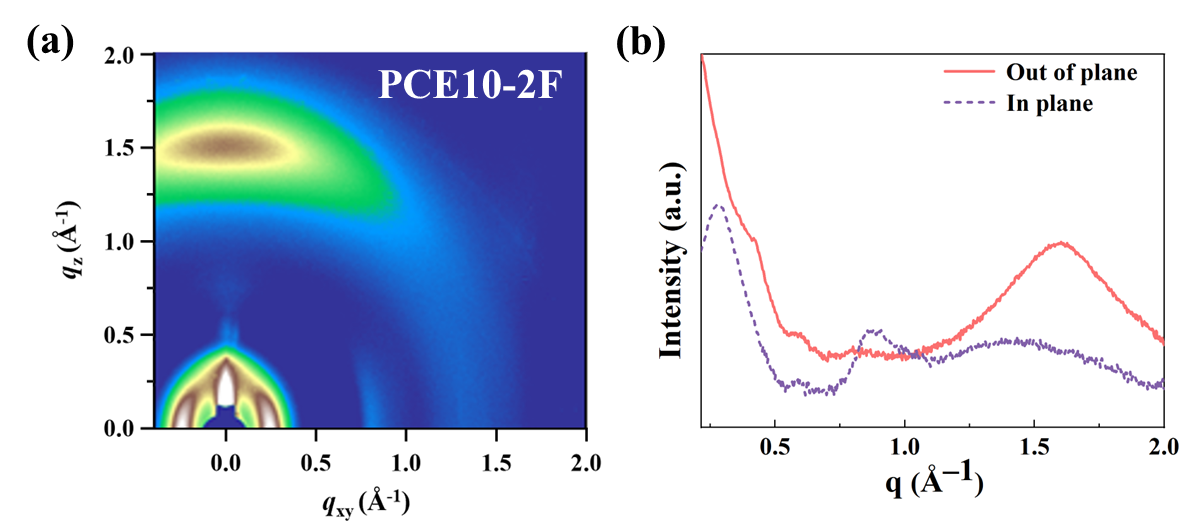
**

**Figure S15.** (a) 2D GIWAXS image of neat PCE10-2F (b) In-plane and out-of-plane line cuts of GIWAXS of the neat PCE10-2F film.


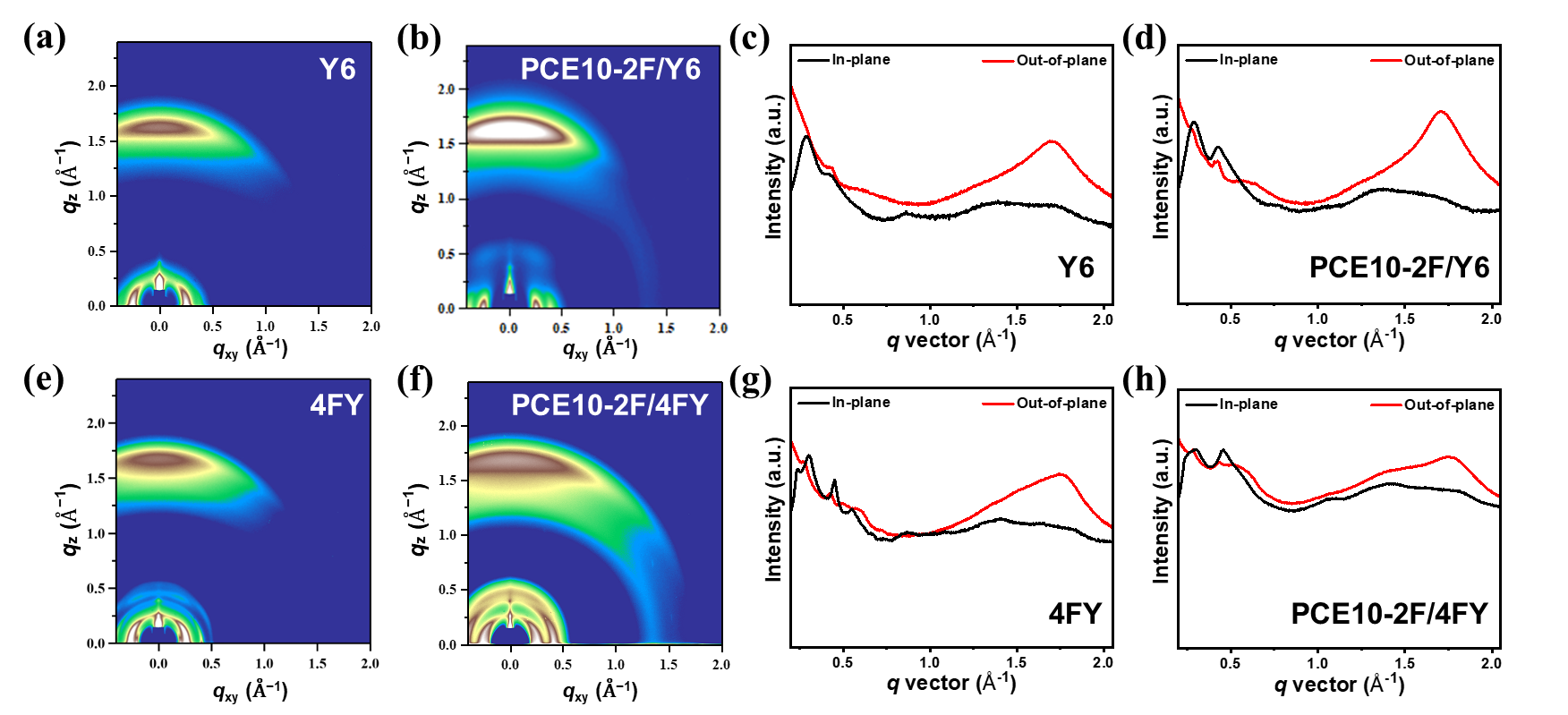


**Figure S16.** 2D GIWAXS images of the neat acceptor (a and e) and blend (b and f) films and In-plane and Out-of-plane line cut profiles of the neat acceptors (c and g) and blend (d and h) films.


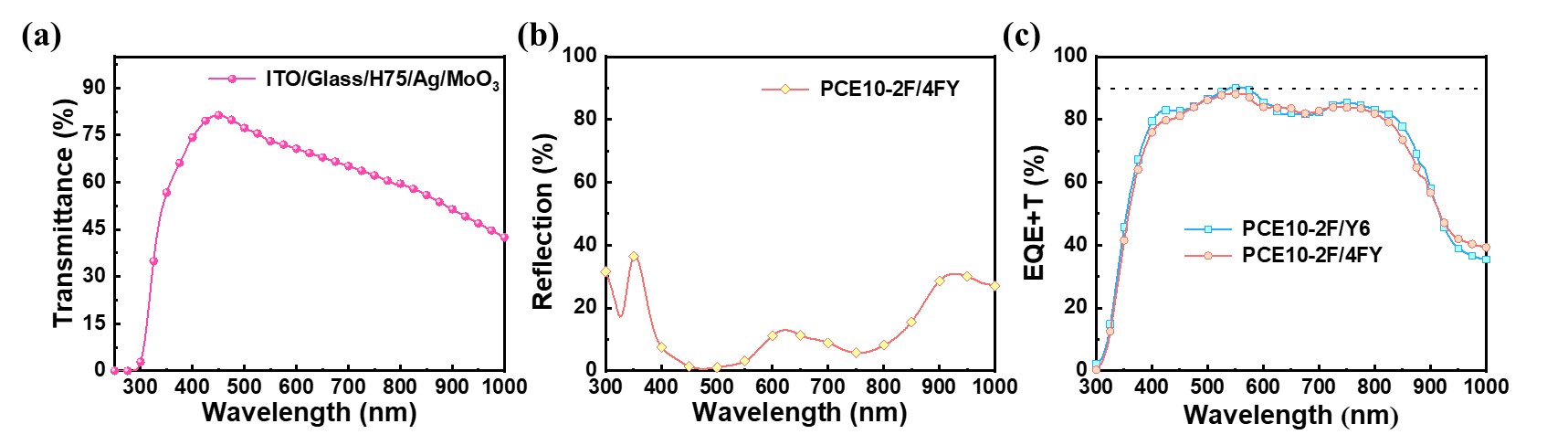


**Figure S17.** (a) The transmittance of the electrodes used in the device. (b) The reflectance spectrum of ST-OSCs. (c) QUE (EQE+T) curves of the ST-OSCs.


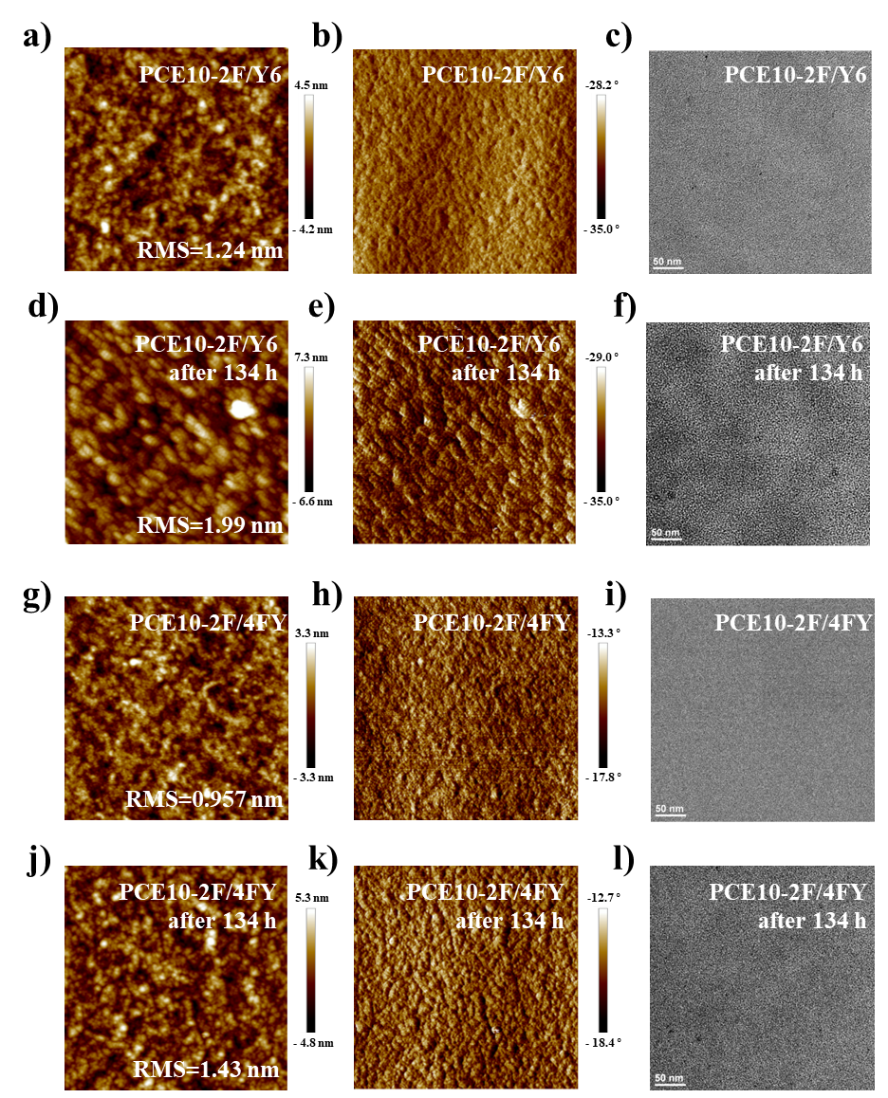


**Figure S18**. AFM surface height (a and d), phase (b and e), and TEM images (c and f) of semitransparent films before and after the PCE10-2F/Y6 diurnal stability test, respectively. AFM surface height (g and j), phase (h and k), and TEM images (i and l) of semitransparent films before and after the PCE10-2F/4FY diurnal stability test, respectively.


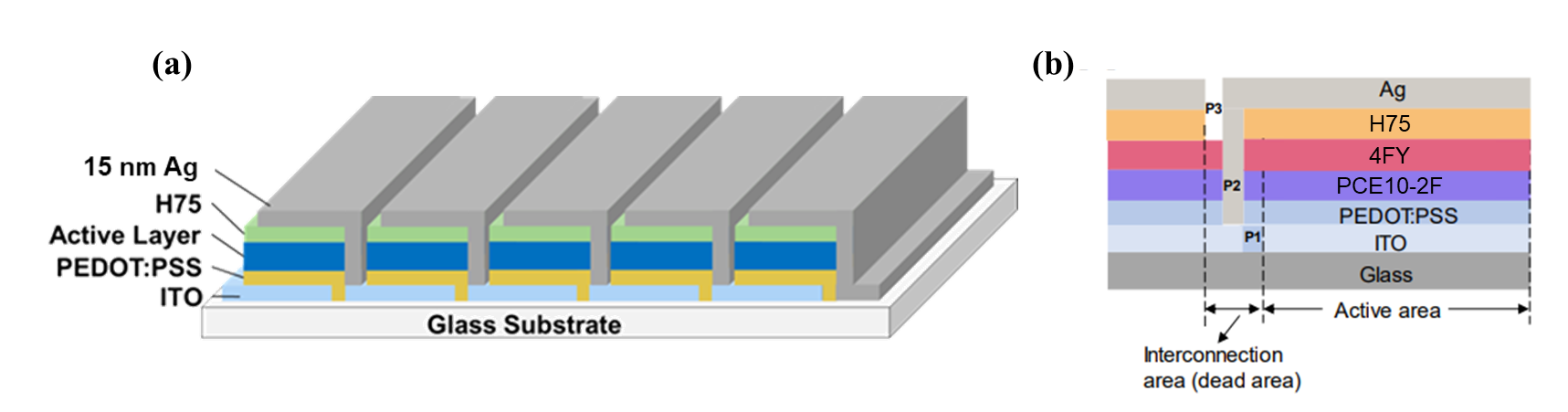


**Figure S19.** (a) Schematic structure and (b) detailed structure of semitransparent module.

**Table S1.** The number of synthetic steps (NSS), reciprocal yield (RY), the number of operation units for the isolation/purification (NUO), the number of column chromatographies for the isolation/purification (NCC), the number of hazardous chemicals (NHC), and synthetic complexity index (SC) for symmetry and asymmetry acceptors.

| Acceptor | | Absolute values | | | | Normalized values | | | | | | SC* (%) |
| --- | --- | --- | --- | --- | --- | --- | --- | --- | --- | --- | --- | --- |
|  |  | NSS | RY | NUO | NCC | NHC | NSS | RY | NUO | NCC | NHC |  |
| Symmetry | Y6 | 14 | 64.8 | 18 | 6 | 64 | 0.74 | 0.13 | 0.64 | 0.6 | 0.69 | 78.12 |
|  | 4FY  (This work) | 16 | 78.9 | 24 | 10 | 71 | 0.84 | 0.16 | 0.86 | 1 | 0.76 | 82.62 |
|  | Y-IC-γe | 13 | 74.5 | 23 | 8 | 57 | 0.68 | 0.15 | 0.82 | 0.8 | 0.61 | 78.06 |
| Asymmetry | BTP-PhC6-C11 | 19 | 486.4 | 28 | 10 | 93 | 1 | 1 | 1 | 1 | 1 | 92.42 |
|  | Y-FIC-γe | 17 | 346.4 | 23 | 8 | 78 | 0.89 | 0.71 | 0.82 | 0.8 | 0.84 | 89.42 |
|  | BTP-S8 | 19 | 333.3 | 24 | 7 | 91 | 1 | 0.69 | 0.86 | 0.7 | 0.98 | 91.62 |

*SC=35NSS/NSSmax+25log(RY)/log(RYmax)+15NCC/NCCmax+15NUO/NUOmax+10NHC/NHCmax

Table S2. Single crystal information of 4FY (CCDC No. 2352496).

| Empirical formula | C_84_H_72_F_6_N_8_O_2_S_5_ |
| --- | --- |
| Formula weight | 1499.84 |
| Temperature/K | 100 |
| Crystal system | monoclinic |
| Space group | C2/c |
| *a*/Å | 24.131(5) |
| *b*/Å | 57.031(11) |
| *c*/Å | 13.464(3) |
| α/° | 90 |
| β/° | 109.30(3) |
| γ/° | 90 |
| Volume/Å^3^ | 17488(7) |
| Z | 8 |
| μ/mm^‑1^ | 0.180 |
| F(000) | 5616.0 |
| Crystal size/mm^3^ | 0.26 × 0.058 × 0.014 |
| Radiation | Synchrotron (*λ* = 0.700) |
| 2Θ range for data collection/° | 1.406 to 48.498 |
| Index ranges | -28 ≤ h ≤ 28, -61 ≤ k ≤ 66, -15 ≤ l ≤ 15 |
| Reflections collected | 28832 |
| Independent reflections | 14538 [*R*_int_ = 0.0486, *R*_sigma_ = 0.0687] |
| Completeness to Θ = 24.25 | 98.1 |
| Data/restraints/parameters | 14538/44/789 |
| Goodness-of-fit on F^2^ | 1.232 |
| Final R indexes [I>=2σ (I)] | *R*_1_ = 0.1156, *wR*_2_ = 0.3424 |
| Final R indexes [all data] | *R*_1_ = 0.1576, *wR*_2_ = 0.3796 |
| Largest diff. peak/hole / e Å^-3^ | 1.30/-0.41 |

**Table S3.** Optical and electrochemical parameters of acceptor Y6 and 4FY.

| Acceptor | λ_film_^max^ (nm) | λ_sol_^max^ (nm) | E_g_^opt^  (eV) | Abs. Coefficient (Film)  (×10^5^ cm^–1^) | Abs. coefficient (solution)  (M^-1^cm^–1^) | HOMO  (eV) | LUMO  (eV) |
| --- | --- | --- | --- | --- | --- | --- | --- |
| Y6 | 828 | 731 | 1.34 | 1.08 | 97.3 | -5.77 | -4.08 |
| 4FY | 811 | 730 | 1.38 | 0.994 | 117.6 | -5.62 | -3.92 |

**Table S4**. Photovoltaic performance of PCE10-2F/4FY based solar cells with different D/A thickness with additive of 0.25%DIO and 0.25%CN.

| D/A | *V_OC_* (V) | *J_SC_* (mA/ cm^2^) | FF (%) | PCE_max_ (%) |
| --- | --- | --- | --- | --- |
| 60nm/40nm | 0.817 | 25.22 | 72.06 | 14.80 |
| 60nm/50nm | 0.801 | 24.91 | 71.55 | 14.26 |
| 50nm/40nm | 0.816 | 24.54 | 72.02 | 14.42 |
| 50nm/50nm | 0.810 | 24.47 | 72.05 | 14.29 |

**Table S5**. Detailed parameter on All NBG-based non-fullerene OSCs devices reported in the literature.

| Acytive layer | *V_OC_* (V) | *J_SC_*  (mA/ cm^2^) | FF  (%) | PCE_max_  (%) | Reference |
| --- | --- | --- | --- | --- | --- |
| PCE10-2F/4FY | 0.817 | 25.20 | 72.06 | 14.80 | This work |
| PTB7-Th: ATT-9 | 0.66 | 30.0 | 67.2 | 13.35 | 1 |
| PCE10-BDT2F-0.8: Y6 | 0.753 | 26.36 | 69.45 | 13.80 | 2 |
| PCE10-2Cl: IT-4F | 0.82 | 18.13 | 71.94 | 10.72 | 3 |
| PTB7-Th: CO*i*8DFIC | 0.69 | 27.3 | 71 | 13.8 | 4 |
| PBTT: IEICO | 0.86 | 17.9 | 61.3 | 9.5 | 5 |
| PBClT: ITIC | 1.01 | 13.95 | 60.05 | 8.46 | 6 |
| PBFTT: IT-4Cl | 0.76 | 19.7 | 73.9 | 11.1 | 7 |
| PTB7-Th: H3 | 0.780 | 25.26 | 67.38 | 13.38 | 8 |
| PL-Cl: F8IC | 0.71 | 26.27 | 69.2 | 12.9 | 9 |
| PCE10: A078 | 0.75 | 24.8 | 0.7 | 13.0 | 10 |
| PTB7-Th: FOIC | 0.743 | 24.0 | 67.1 | 12.0 | 11 |
| PTB7-Th: FOIC: PC_71_BM | 0.753 | 23.83 | 66.5 | 12.32 | 11 |
| PCE-10: BT-CIC: TT-FIC | 0.68 | 23.3 | 72 | 11.4 | 12 |
| PTB7-Th: IEICO-4F | 0.712 | 27.3 | 65.7 | 12.8 | 13 |
| PTB7-Th: IEICO-4Cl | 0.727 | 22.8 | 62 | 10.3 | 14 |
| PTB7-Th: IUIC | 0792 | 21.51 | 64.7 | 11.2 | 15 |
| PTB7-Th: COi8DFIC: IEICO-4F | 0.714 | 23.97 | 69.78 | 11.94 | 16 |
| PTB7-Th: ACS8 | 0.75 | 25.3 | 69.3 | 13.2 | 17 |
| PCE10: ICBA: Y8 | 0.742 | 23.51 | 73.74 | 12.84 | 18 |
| PTB7-Th: FNIC1 | 0.741 | 23.93 | 73.4 | 13.0 | 19 |
| PTB7-Th: FNIC2 | 0.774 | 19.97 | 66.4 | 10.3 | 19 |
| PTB7-Th: ATT-2 | 0.73 | 20.75 | 63 | 9.58 | 20 |
| PCE10-2F/Y6 | 0.789 | 26.14 | 70.32 | 14.53 | 21 |
| PM2:Y6-BO | 0.72 | 22.1 | 70.6 | 11.1 | 22 |
| PTB7-Th: BTPSeV-4F | 0.66 | 30.1 | 71.4 | 14.2 | 23 |
| DA:PCE10-2F/Y6:Y5 | 0.812 | 26.2 | 73.22 | 15.57 | 24 |

**Table S6.** Detailed energy loss of opaque devices based on PCE10-2F/Y6 and PCE10-2F/4FY.

| **Active Layer** | ***E*_g_**  **[eV]** | ***qV_OC_***  **[eV]** | ***qVoc^SQ^***  **[eV]** | ***qVoc^rad^***  **[eV]** | **△*E*_1_**  **[eV]** | **△*E*_2_**  **[eV]** | **△*E*_3_**  **[eV]** | ***E*_loss_**  **[eV]** | **EQE_EL_**  **(%)** |
| --- | --- | --- | --- | --- | --- | --- | --- | --- | --- |
| **PCE10-2F/Y6** | 1.387 | 0.793 | 1.126 | 1.070 | 0.261 | 0.056 | 0.277 | 0.594 | 2.27×10^-3^ |
| **PCE10-2F/4FY** | 1.393 | 0.816 | 1.132 | 1.086 | 0.261 | 0.046 | 0.270 | 0.577 | 2.87×10^-3^ |

where *E*_g_ is the bandgap, *q* is the elementary charge, *qVoc^SQ^* is the maximum voltage based on the Shockley-Queisser limit (SQ limit), *qVoc^rad^* is the open-circuit voltage when there is only radiative recombination.

**Table S7**. Hole and electron mobilities of PCE10-2F/Y6 and PCE10-2F/4FY devices in the dark.

| **Device** | ***μ*_h_(cm^2^ V^−1^ s^−1^)** | ***μ*_e_(cm^2^ V^−1^ s^−1^)** | | ***μ*_h_/*μ*_e_** |
| --- | --- | --- | --- | --- |
| PCE10-2F/Y6 | 8.729×10^-4^ | 8.211×10^-4^ | 1.063 | |
| PCE10-2F/4FY | 1.013×10^-3^ | 9.721×10^-4^ | 1.042 | |

**Table S8**. Operating characteristics of opaque OSC devices (D/A thickness of 45 nm/40 nm) under simulated AM 1.5G, 100 mW cm^−2^ illumination.

| **Devices** | ***V_OC_* (V)** | ***J_SC_***  **(mA/ cm^2^)** | ***J_SC cal_***  **(mA/ cm^2^)** | **FF**  **(%)** | **PCE_max_**  **(%)** | **AVT of active layer (%)** |
| --- | --- | --- | --- | --- | --- | --- |
| PCE10-2F/Y6 | 0.795 | 24.54 | 23.56 | 71.13 | 13.88 | 64.75 |
| PCE10-2F/4FY | 0.817 | 23.57 | 22.48 | 74.95 | 14.45 | 64.86 |

**Table S9**. Summarized GIWAXS parameters for the ordering structures of neat films and LBL blend films.

| Materials | Out-of-Plane | | | | In-Plane | | | |
| --- | --- | --- | --- | --- | --- | --- | --- | --- |
|  | π-π stacking peak | | | | (110) peak | | | |
|  | q (Å^-1^) | d-spacing  (Å) | FWHM (Å^-1^) | Coherence length (Å) | q (Å^-1^) | d-spacing  (Å) | FWHM (Å^-1^) | Coherence length (Å) |
| PCE10-2F | 1.597 | 3.936 | 0.28 | 20.379 | 0.282 | 22.313 | 0.079 | 71.729 |
| Y6 | 1.703 | 3.689 | 0.197 | 29.092 | 0.288 | 21.797 | 0.067 | 84.63 |
| 4FY | 1.746 | 3.599 | 0.288 | 25.087 | 0.297 | 21.18 | 0.052 | 108.472 |
| PCE10-2F/Y6 | 1.710 | 3.675 | 0.168 | 34.011 | 0.289 | 21.759 | 0.065 | 86.639 |
| PCE10-2F/4FY | 1.766 | 3.557 | 0.161 | 35.613 | 0.296 | 21.208 | 0.097 | 58.155 |

**Table S10**. Detailed parameters on ST-OSM devices more than active area of 10cm^2^ without complex optical control.

|  | Active layer | *V*_OC_  (V) | *J*_SC_  (mA/cm^2^) | FF  (%) | PCE  (%) | AVT  (%) | Area  (cm^2^) | LUE | Ref. |
| --- | --- | --- | --- | --- | --- | --- | --- | --- | --- |
| All-NBG | PCE10-2F/4FY (This work) | 3.92 | 2.48 | 69.69 | 6.78 | 45.43 | 18 | 3.10 | This work |
|  | PTB7-Th:IEICO-4F | - | - | - | 2.31 | 70.6 | 10.3 | 1.63 | 25 |
|  | PTB7-Th:IEICO-4F | - | - | - | 1.12 | 80.6 | 10.3 | 0.90 | 25 |
|  | PTB7-Th:IEICO-4F | 5.67 | 1.2 | 52 | 3.5 | 63 | 56 | 2.2 | 26 |
|  | PCE-10:BT-CIC | 2.0 | Isc 51.1 mA | 63 | 7.2 | 38.1 | 9 | 2.74 | 27 |
| WBG | PBDTTT-EFT:PCBM | 12.49 | 0.76 | 47 | 4.50 | 10.00 | 216 | 0.45 | 28 |
|  | PBDTTT-EFT:PCBM | 12.04 | 0.70 | 49 | 4.09 | 10.00 | 216 | 0.45 | 28 |
|  | pDPP5T-2:PCBM | 9.10 | 0.41 | 62.7 | 2.34 | 52.00 | 64 | 1.21 | 29 |
|  | PM6:BTP-BOCl12 | 5.65 | 3.07 | 68.9 | 12.01 | 22.3 | 25 | 2.67 | 30 |
|  | PM6/BTP-4Cl-12 | 6.20 | 2.9 | 48.9 | 8.8 | 22 | 30 | 1.94 | 31 |
|  | PM6:DTY6 | 4.81 | 3.60 | 66.8 | 11.6 | 18 | 10.8 | 2.09 | 32 |
|  | P3HT:IDTBR | 7.11 | 10.87 | 64.9 | 5.00 | 6.20 | 59.52 | 0.31 | 33 |
|  | PBTZT-stat-BDTT-8:PCBM | 7.0 | 119.7 | 62 | 4.5 | 20 | 114.5 | 0.9 | 34 |
|  | PBDT-T-2F/L8-BO | 2.54 | 6.18 | 67 | 9.54 | 21.2 | 10.8 | 2.0 | 35 |
|  | PM6/BTP-4Cl-12 | 6.6 | 2.5 | 43.8 | 7.2 | 22.3 | 30 | 1.6 | 36 |
|  | PTQ10:BTP-4F-12:PC71BM | 6.8 | 2.29 | 62.7 | 9.75 | 11.3 | 10.17 | 1.1 | 37 |
|  | PM6:BTP-eC9:L8-BO | 13.15 | 0.82 | 53.5 | 5.77 | 37.5 | 30.72 | 2.16 | 38 |
|  | PV-X | 5.22 | 2.25 | 49.6 | 5.9 | 28.3 | 60 | 1.67 | 39 |
|  | PBTZT-stat-BDTT-8:PCBM | 0.80 | 10.00 | 60 | 4.80 | 20.00 | 114.5 | 0.96 | 40 |
|  | PTzBI-Cl: DT-Y6:BTR-Cl | 5.6 | 2.9 | 65.9 | 10.2 | 32.8 | 21 | 3.3 | 41 |
|  | PTzBI-Cl: DT-Y6:BTR-Cl | 10.7 | 1.2 | 49.1 | 6.7 | 32.9 | 100 | 2.2 | 41 |
|  | PTB7:PC_70_BM | - | - | - | 8.22 | 41.45 | 18.63 | 3.4 | 42 |

**REFERENCES**

[1] W. Liu, S. Sun, S. Xu, H. Zhang, Y. Zheng, Z. Wei and X. Zhu *Adv. Mater*., 2022, **18**, 2200337.

[2] X. Huang, L. Zhang, Y. Cheng, J. Oh, C. Li, B. Huang, L. Zhao, J. Deng, Y. Zhang, Z. Liu, F. Wu, X. Hu, C. Yang, L. Chen, Y. Chen, *Adv. Funct. Mater*. 2022, **32**, 2108634.

[3] X. Huang, J. Oh, Y. Cheng, B. Huang, S. Ding, Q. He, F. Wu, C. Yang, L. Chen and Y. Chen, *J. Mater. Chem*. A, 2021, **9**, 5711-5719.

[4] W. Li, M. Chen, J. Cai, E. L.K. Spooner, H. Zhang, R. S. Gurney, D. Liu, Z. Xiao, D. G. Lidzey, L. Ding and T. Wang, *Joule*, 2019, **3**, 819-833.

[5] G. Li, W. Li, X. Guo, B. Guo, W. Su, Z. Xu, M. Zhan, *Org. Electron.*, 2019, **64**, 241-246.

[6] P. Chao, Z. Mu, H. Wang, D. Mo, H. Chen, H. Meng, W. Chen and F. He, *ACS Applied Energy Materials*, 2018, **1**, 2365-2372.

[7] W. Su, Q. Fan, X. Guo, J. Wu, M. Zhang and Y. Li, *Phys. Chem. Chem. Phys*., 2019, **21**, 10660-10666.

[8] Y. Li, C. He, L. Zuo, F. Zhao, L. Zhan, X. Li, R. Xia, H.L. Yip, Li, C.Z., X. Liu, and H. Chen, *Adv. Energy Mater*., 2021,**11**, 2003408.

[9] Y. Chang, X. Zhu, L. Zhu, Y. Wang, C. Yang, X. Gu, Y. Zhang, J. Zhang, K. Lu, X. Sun and Z. Wei, *Nano Energy*, 2021, **86**, 106098.

[10] Y. Li, X. Guo, Z. Peng, B. Qu, H. Yan, H. Ade, M. Zhang and S. R. Forrest, *P.N.A.S.* 2020, **117**, 21147.

[11] T. Li, S. Dai, Z. Ke, L. Yang, J. Wang, C. Yan, W. Ma, and X. Zhan, *Adv. Mater*. 2018, **30**, 1705969.

[12] Y. Li, C. Ji, Y. Qu, X. Huang, S. Hou, C.Z. Li, L.S. Liao, L.J. Guo and S.R. Forrest, *Adv. Mater*., 2019, **31**, 1903173.

[13] X. Song, N. Gasparini, L. Ye, H. Yao, J. Hou, H. Ade and D. Baran, *ACS Energy Lett.*, 2018, **3**, 669-676.

[14] Y. Cui, C. Yang, H. Yao, J. Zhu, Y. Wang, G. Jia, F. Gao and J. Hou, *Adv. Mater*., 2017, **29**, 1703080. 2.

[15] B. Jia, S. Dai, Z. Ke, C. Yan, W. Ma and X. Zhan, *Chem. Mater*., 2017, **30**, 239-245.

[16] X. Ma, Z. Xiao, Q. An, M. Zhang, Z. Hu, J. Wang, L. Ding and F. Zhang, *J. Mater. Chem. A,* 2018, **6**, 21485-21492.

[17] J. Chen, G. Li, Q. Zhu, X. Guo, Q. Fan, W. Ma, and M. Zhang, *J. Mater. Chem. A*, 2019, **7**, 3745-3751.

[18] C. Zhu, H. Huang, Z. Jia, F. Cai, J. Li, J. Yuan, L. Meng, H. Peng, Z. Zhang, Y. Zou, and Y Li., *Sol Energy,* 2020, **204**, 660-666.

[19] J. Wang, J. Zhang, Y. Xiao, T. Xiao, R. Zhu, C. Yan, Y. Fu, G. Lu, X. Lu, S. R. Marder and X. Zhan, *J. Am. Chem. Soc*., 2018, **140**, 9140-9147.

[20] F. Liu, Z. Zhou, C. Zhang, J. Zhang, Q. Hu, T. Vergote, F. Liu, T.P. Russell, and X. Zhu, *Adv. Mater*., 2017, **29**, 1606574.

[21] Xuexiang Huang, Yujun Cheng, Yuan Fang, Lifu Zhang, Xiaotian Hu, Sang Young Jeong, Hean Zhang, Han Young Woo, Feiyan Wu, and Lie Chen, *Energy Environ. Sci.*, 2022, **15**, 4776-4788

[22] T. Jiang, G. Zhang, R. Xia, J. Huang, X. Li, M. Wang, H.-L. Yip, Y. Cao, *Materials Today Energy*, 2021, 21, 100807.

[23] Zhenrong Jia, Qing Ma, Zeng Chen, Lei Meng, Nakul Jain, Indunil Angunawela, Shucheng Qin, Xiaolei Kong, Xiaojun Li, Yang (Michael) Yang, Haiming Zhu, Harald Ade, Feng Gao, and Yongfang Li, *Nat. Commun*., **14**, 1236 (2023).

[24] Xuexiang Huang, Xinyuan Ren, Yujun Cheng, Youhui Zhang, Zhe Sun, Sangjin Yang, Seoyoung Kim, Changduk Yang, Feiyan Wu, and Lie Chen,
*Energy Environ. Sci.*, 2024, **17**, 2825-2836.

[25] Ruiqian Meng, Qiangqing Jiang, Dianyi Liu, *npj Flex Electron*, 2022, **6**, 39.

[26] Anirudh Sharma, Nicola Gasparini, Anastasia Markina, Safakath Karuthedath, Julien Gorenflot, Han Xu, Jianhua Han, Ahmed Balawi, Wenlan Liu, Daniel Bryant, Jules Bertrandie, Joel Troughton, Sri Harish Kumar Paleti, Helen Bristow, Frederic Laquai, Denis Andrienko, and Derya Baran, *Adv. Mater.,* 2024, *36*, 2305367

[27] Kuan‐Min Huang, Ying Qian Wong, Man‐Chun Lin, Chao‐Hsuan Chen, Chung‐Hung Liao, Jen‐Yueh Chen, Yuan‐Han Huang, Yu‐Fan Chang, Pei‐Ting Tsai, Szu‐Han Chen, Ching‐Ting Liao, Yu‐Cih Lee, Ling Hong, Chih‐Yu Chang, Hsin‐Fei Meng, Ziyi Ge, Hsiao‐Wen Zan, Sheng‐Fu Horng, Yu‐Chiang Chao, Hin Yong Wong, *Prog Photovolt Res Appl*., 2019, **27**, 264–274.

[28] Fei Guo, Peter Kubis, Thomas Przybilla, Erdmann Spiecker, Andre Hollmann, Stefan Langner, Karen Forberich, and Christoph J. Brabec, *Adv. Energy Mater*. 2015, **5**, 1401779.

[29] Xinjing Huang, Dejiu Fan, Stephen R. Forrest, *Org. Electron.*, 2021, **97**, 106276.

[30] J.Y. Fan, Z.X. Liu, J. Rao, K. Yan, Z. Chen, Y. Ran, B. Yan, J. Yao, G. Lu, H. Zhu, C.Z. Li, H. Chen, *Adv. Mater*. 2022, **34**, 2110569.

[31] Y.W. Han, H.S. Lee, D.K. Moon, *ACS Appl. Mater. Interfaces.*, 2021, 13, 19085–19098.

[32] S. Dong, T. Jia, K. Zhang, J. Jing, F. Huang, *Joule*., 2020, 4, 2004–2016.

[33] S. Strohm, F. Machui, S. Langner, P. Kubis, N. Gasparini, M. Salvador, I. McCulloch, H.J. Egelhaaf, C.J. Brabec, *Energy Environ. Sci*., 2018, 11, 2225–2234.

[34] S. Berny, N. Blouin, A. Distler, H.J. Egelhaaf, M. Krompiec, A. Lohr, O.R. Lozman, G.E. Morse, L. Nanson, A. Pron, T. Sauermann, N. Seidler, S. Tierney, P. Tiwana, M. Wagner, H. Wilson, *Adv. Sci*., 2015, 3, 1500342.

[35] Wusong Zha, Li-Min Chen, Shaoming Sun, Xiaomei Gao, Yunfei Han, Tong Liu, Qun Luo, Yu-Chiang Chao, Hsiao-Wen Zan, Hsin-Fei Meng, Xiaozhang Zhu, and Chang-Qi Ma, *Sol. RRL*, 2023, **7**, 2300322

[36] Yong Woon Han, Hyoung Seok Lee, and Doo Kyung Moon, *ACS Appl. Mater. Interfaces* 2021, **13**, 19085−19098

[37] Josua Wachsmuth, Andreas Distler, Chao Liu, Thomas Heumüller, Yang Liu, Catherine M. Aitchison, Alina Hauser, Michael Rossier, Amélie Robitaille, Marc-Antoine Llobel, Pierre-Olivier Morin, Anaïs Thepaut, Charline Arrive, Iain McCulloch, Yinhua Zhou, Christoph J. Brabec, and Hans-Joachim Egelhaaf, *Sol. RRL,* 2023, **7**, 2300602.

[38] Mengan Zhao, Jiang Wu, Hao Tang, Xueting Yi, Zekun Liu, Minghui Huang, Yingying Fu, and Zhiyuan Xie, *Adv. Optical Mater.,* 2024, 2401776

[39] Yun-Ming Sung, Cheng-Si Tsao, Hou-Chin Cha, and Wei-Yang Ma, *Prog Photovolt Res Appl*., 2023, **31**, 803–812.

[40] F. Guo, P. Kubis, T. Przybilla, E. Spiecker, A. Hollmann, S. Langner, K. Forberich, C.J. Brabec, *Adv. Energy Mater*., 2015, 5, 1401779.

[41] Juxuan Xie, Ju Zhao, Zhisheng Zhou, Kai Zhang, Jiangkai Yu, Chang Zhu, and Fei Huang, *Energy Environ. Sci*., 2024,17, 7681-7690.

[42] Jae Hoon Jeong, Muhammad Jahandar, Adi Prasetio, Jeong Min Kim, Jung Hyun Kim, Soyeon Kim, Dong Chan Lim, *Chem. Eng. J.*, 2021, 419, 129672
